# Ectopic expression of *Triticum polonicum VRT-A2* underlies elongated glumes and grains in hexaploid wheat in a dosage-dependent manner

**DOI:** 10.1093/plcell/koab119

**Published:** 2021-05-01

**Authors:** Nikolai M. Adamski, James Simmonds, Jemima F. Brinton, Anna E. Backhaus, Yi Chen, Mark Smedley, Sadiye Hayta, Tobin Florio, Pamela Crane, Peter Scott, Alice Pieri, Olyvia Hall, J. Elaine Barclay, Myles Clayton, John H. Doonan, Candida Nibau, Cristobal Uauy

**Affiliations:** 1 John Innes Centre, Norwich Research Park, Norwich, NR4 7UH, UK; 2 The National Plant Phenomics Centre, Institute of Biological, Rural and Environmental Sciences (IBERS), Aberystwyth University, Gogerddan, Aberystwyth, SY23 3EE, UK

## Abstract

Flower development is an important determinant of grain yield in crops. In wheat (*Triticum* spp.), natural variation for the size of spikelet and floral organs is particularly evident in *Triticum turgidum* ssp. *polonicum* (also termed *Triticum polonicum*), a tetraploid subspecies of wheat with long glumes, lemmas, and grains. Using map-based cloning, we identified *VEGETATIVE TO REPRODUCTIVE TRANSITION 2* (*VRT2*), which encodes a MADS-box transcription factor belonging to the SHORT VEGETATIVE PHASE family, as the gene underlying the *T. polonicum* long-glume (*P1*) locus. The causal *P1* mutation is a sequence rearrangement in intron-1 that results in ectopic expression of the *T. polonicum VRT-A2* allele. Based on allelic variation studies, we propose that the intron-1 mutation in *VRT-A2* is the unique *T. polonicum* subspecies-defining polymorphism, which was later introduced into hexaploid wheat via natural hybridizations. Near-isogenic lines differing for the *P1* locus revealed a gradient effect of *P1* across spikelets and within florets. Transgenic lines of hexaploid wheat carrying the *T. polonicum VRT-A2* allele show that expression levels of *VRT-A2* are highly correlated with spike, glume, grain, and floral organ length. These results highlight how changes in expression profiles, through variation in cis-regulation, can affect agronomic traits in a dosage-dependent manner in polyploid crops.

## Teaser

An intron-1 rearrangement in the MADS-box transcription factor gene *VRT-A2* leads to its misexpression and defines the long-glume phenotype of Polish wheat (*T. polonicum*).

## Introduction

**Figure 1:**
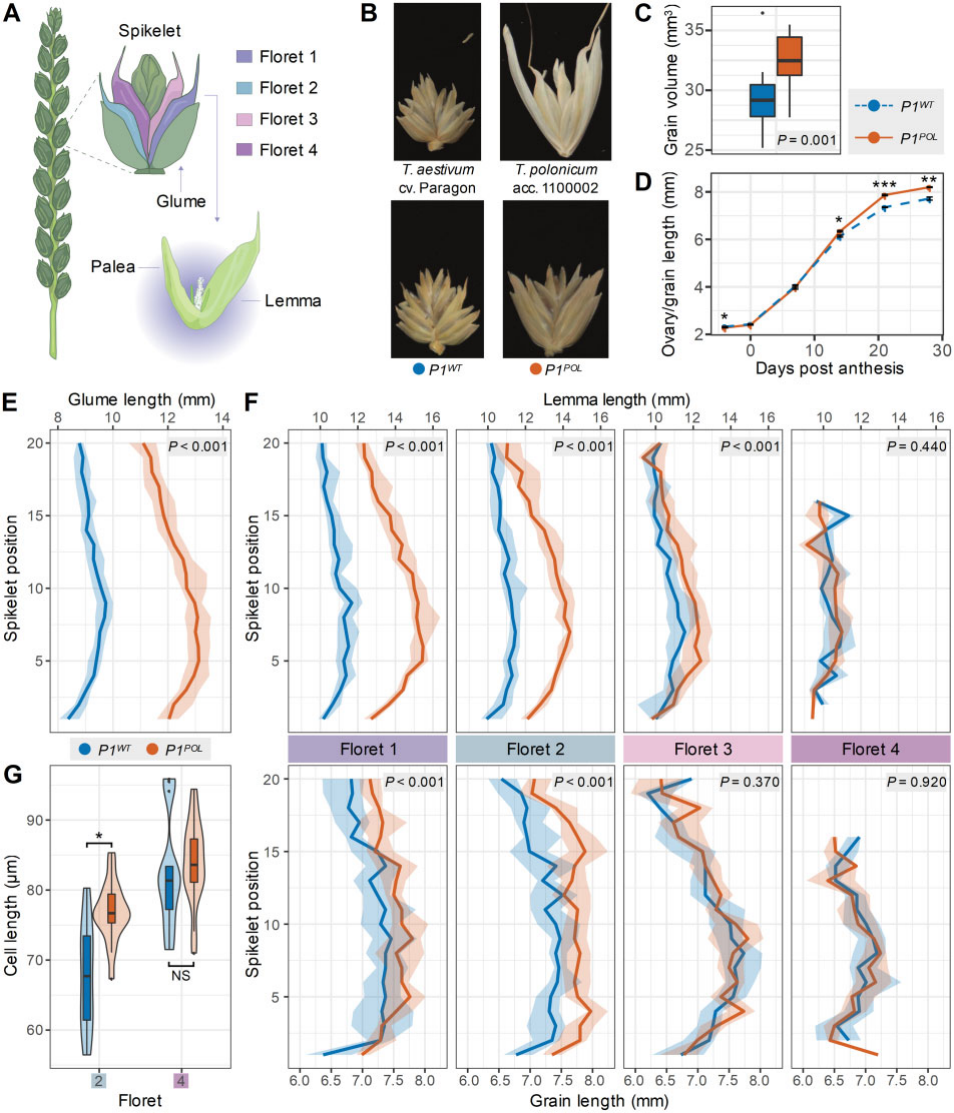
Phenotypic effects of *P1* in NILs. A, Drawing of a wheat spike, with a close-up of an individual spikelet. The first four florets on the spikelet are color coded. A close-up of an open floret depicts its two enveloping sheathing structures (lemma and palea). B, Spikelets of the parental hexaploid bread wheat cultivar “Paragon” and tetraploid *T. polonicum* accession 11000002, and the *P1^WT^* and *P1^POL^* NILs. C, Grain volume was measured using a CT-scanner to image field-grown spikes of the two NILs (*n* = 15 grains). D, Timecourse tracking ovary/grain length development in field-grown *P1^WT^* and *P1^POL^* NILs (*n* = 50 spikes per timepoint). E, Glume length along spikes of *P1^WT^* and *P1^POL^* NILs; positions are numbered from basal (position 0) to apical (position 20) spikelets (*n* = 15 spikes). F, Lemma and grain length at each floret position along *P1^WT^* and *P1^POL^* NILs spikes. Spikelet positions as in E (*n* = 15 spikes). In (E) and (F) bold line represents the median value, ribbon represents the interquartile range. G, Pericarp cell length from middle sections of grains from florets 2 and 4 for the *P1^WT^* and *P1^POL^* NILs (*n* = 18 grains). In (C) and (G), the box represents the middle 50% of data with the borders of the box representing the 25th and 75th percentile. The horizontal line in the middle of the box represents the median. Whiskers represent the minimum and maximum values, unless a point exceeds 1.5 times the inter-quartile range in which case the whisker represents this value and values beyond this are plotted as single points (outliers). See Supplemental Data Set S6 for additional measurements. Error bars represent mean ± sem. *P < 0.05; **P < 0.01; ***P < 0.001

The genus *Triticum* contains multiple wheat subspecies exhibiting traits of agronomic interest, making them valuable genetic resources for breeding. Among these, *Triticum turgidum* ssp. *polonicum* (Polish wheat, referred to here as *Triticum polonicum*), a tetraploid (AABB) spring wheat, is characterized by elongated glumes and grains, the latter of which is an important component of crop yield. Glumes are sterile bract-like organs that subtend spikelets, which are lateral branches that contain several grain-producing florets. Each floret is composed of two leaf-like sheathing structures, the lemma and the palea, as well as two lodicules, three stamens and a pistil (Figure 1A).

**Figure:**
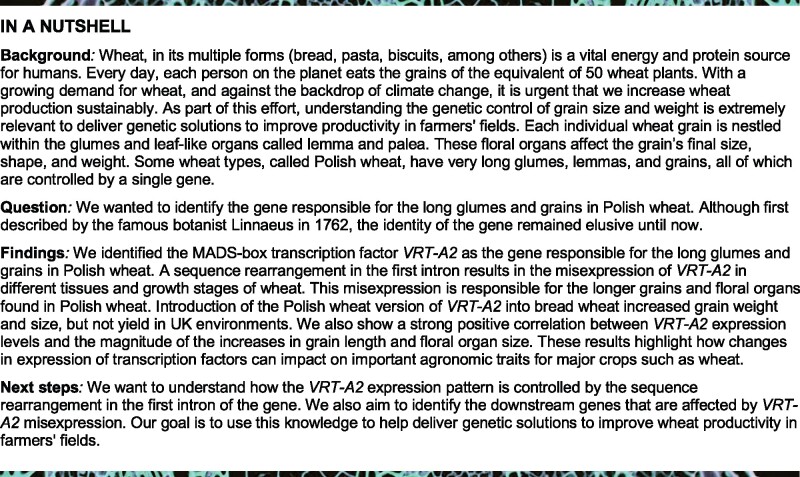


It was established over 100 years ago that glume length in *T. polonicum* is controlled by a single locus (Biffen, 1905; Engledow, 1920). The *P* or *P1* locus (from Polish wheat) was mapped to chromosome 7A (Matsumura, 1950) and subsequent studies refined the map location to the short arm of chromosome 7A (Watanabe et al., 1996; Kosuge et al., 2010; Okamoto and Takumi, 2013). While *T. polonicum* as a subspecies is defined by its highly elongated glumes, Biffen (1905), Engledow (1920), and Okamoto and Takumi (2013) also observed that the long-glume trait was completely linked with elongated grains, suggesting pleiotropic effects of the *P1* locus. Okamoto and Takumi (2013) further showed that the *T. polonicum P1* allele was also linked to an increase in spike length and a reduction in the number of spikelets per spike. These studies all determined a semi-dominant effect of *P1*, with heterozygous lines being intermediate to the parents for both glume and grain length.

In addition to tetraploid *T. polonicum*, there are a number of hexaploid bread wheat accessions with elongated glumes. These include the Chinese landrace *T. petropavlovskyi* (also called “Daosuimai” or rice-head wheat) as well as members of the Portuguese landrace group “Arrancada”. It is hypothesized that the long-glume phenotype of these hexaploid wheat accessions is the result of natural hybridization between *T. polonicum* and *T. aestivum* landraces (Chen et al., 1985, 1988; Watanabe and Imamura, 2002; Akond and Watanabe, 2005; Akond et al., 2008; Kang et al., 2010; Chen et al., 2013). Indeed, for both *T. petropavlovskyi* and “Arrancada”, the causal genetic locus for long glumes was mapped to chromosome 7A, supporting the hypothesis of a shared origin with *T. polonicum* (Watanabe and Imamura, 2002; Watanabe et al., 2004).

The spatial and temporal expressions of MADS-box transcription factors determine floral organ identity and developmental phase transitions in plants. The *Tunicate1* mutant of maize (*Zea mays*), known as pod corn, exhibits highly elongated leaf-like glumes that cover the kernels (Mangelsdorf and Galinat, 1964; Langdale et al., 1994). Genetic studies identified the causal gene as *Z. mays MADS19* (*ZMM19*), which encodes a member of the *SHORT VEGETATIVE PHASE* (*SVP*) gene family of MADS-box transcription factors (Han et al., 2012; Wingen et al., 2012). A rearrangement in the promoter region of *ZMM19* causes its ectopic expression, which leads to the dosage-dependent phenotype (Han et al., 2012; Wingen et al., 2012). Ectopic expression of *ZMM19* in *Arabidopsis thaliana* leads to enlarged sepals, suggesting a conserved mechanism (Wingen et al., 2012).

Spikelet morphology and organ size are tightly correlated with final grain weight in wheat (Millet, 1986). Despite their importance, we have relatively little understanding of the genes controlling spikelet and floral organ size in wheat. Here, we characterized the *P1* locus of *T. polonicum*, which has pleiotropic effects on glume, floral organ, and grain size. We show that the *P1* long-glume phenotype is due to the ectopic expression of *VEGETATIVE TO REPRODUCTIVE TRANSITION 2* (*VRT-A2*), an *SVP* MADS-box transcription factor. The ectopic expression of *VRT-A2* is due to a sequence rearrangement in the first intron, which defines *T. polonicum* as a subspecies. Expression levels of *VRT-A2* affect spike, glume, grain, and floral organ length in a dosage-dependent manner.

## Results

### The long-glume *T. polonicum P1* allele enhances grain weight through longer grains

To evaluate the performance of the *T. polonicum P1* allele, we developed BC_4_ and BC_6_ near isogenic lines (NILs) by crossing *T. polonicum* accession 1100002 to the hexaploid spring wheat cultivar Paragon (Table 1 andFigure 1B). We verified the isogenic status of these lines using the Breeders’ 35K Axiom Array (Supplemental Figure S1; Allen et al., 2017) and assessed the *T. polonicum* (*P1^POL^*) and wild-type (*P1^WT^*) NILs in the field over multiple years and environments. The *P1^POL^* NILs had longer glumes and lemmas than wild-type Paragon NILs (Figure 1B) and were on average 6 cm taller due to an increase in peduncle (final internode) and spike lengths (1.6 cm; *P *<* *0.01; Table 1;Supplemental Data Sets S1–S3 and Supplemental Figure S2). The increase in spike length, alongside a minor increase in spikelet number (0.4 spikelets per spike; *P *=* *0.06), led to a 11.3% decrease in spikelet density (spikelets per cm) in *P1^POL^* with respect to *P1^WT^* NILs (significant in three of four environments; Supplemental Data Set S1). The *P1^POL^* NILs also flowered on average 0.8 days later (*P *<* *0.001) than the wild-type NILs. We observed consistent positive effects on thousand grain weight (TGW) in *P1^POL^* (5.5%; *P *<* *0.001), which were driven by significant increases in grain length (5.0%; *P *<* *0.001), but not grain width (Table 1;Supplemental Data Sets S1 and S4). The increase in grain length resulted in an increased grain volume (10.7%; *P *=* *0.001) as determined by CT scans of a single year of field samples (Figure 1C;Supplemental Data Set S4). The increased TGW in *P1^POL^* NILs also translated into a significant increase in hectoliter weight in four out of five environments (2.3%; *P *<* *0.01). Final yield, however, was not significantly different between NILs despite the increase in TGW (Table 1;Supplemental Data Set S1).

**Table 1:** Phenotypic effects of the *P1* allele in Paragon NILs. *P1^POL^* effect is the percentage difference (except height and spike length in cm; heading date in days) between the *P1^WT^* and the *P1^POL^* NILs

Allele	Height (cm)	Spike length (cm)	Heading date (days)	HLW (kg·hl^-1^)	TGW (g)	Grain width (mm)	Grain length (mm)	Yield (kg·plot^-1^)
*P1^POL^*	90.6	13.2	218.5	77.2	46.8	3.584	6.757	5.237
*P1^WT^*	84.6	11.6	217.7	75.5	44.4	3.589	6.438	5.212
*P1^POL^* effect	6.0	1.6	0.8	2.3%	5.5%	−0.1%	5.0%	0.5%
*P* value	2.0E−13	0.005	4.4E−07	0.009^a^	6.9E-06	*Interact.*	< 2.2E−16	NS

The *P-*value of the ANOVA main effect is presented, apart from grain width, which had a significant interaction across environments (simple effects and detailed breakdown in Supplemental Data Set S1). Values represent means of six field experiments (except spike length *n* = 4)

a *P1^POL^* NIL was significant in five out of six environments.

### The *P1* allele from *T. polonicum* enhances grain size in florets 1 and 2 through an increase in cell length

We conducted more in-depth phenotyping to identify the first time point during grain development in which differences in grain length are established between *P1* NILs. We dissected and measured field-grown grain samples from florets 1 and 2 of five central spikelets from *P1^WT^* and *P1^POL^* NILs at six timepoints during grain development (Figure 1D; second year data in Supplemental Figure S3). We did not detect consistent differences in ovary length before and at anthesis nor in grain length 7 days post anthesis (dpa). However, at 14 dpa, grains from *P1^POL^* NILs were 3.4% longer than grains from *P1^WT^* NILs (*P *<* *0.05; Supplemental Data Set S5). The increased grain length in *P1^POL^* NILs was maintained at 21 and 28 dpa (7.0% and 6.2% longer grains than *P1^WT^*, respectively; *P *<* *0.002; Supplemental Data Set S5). These results, consistent in two independent field seasons (Supplemental Figure S3), suggest that the difference in grain length between *P1* NILs is established during mid-grain filling.

We next measured the size (length, width, and area) of glumes, floral organs (lemma and palea), and grains across spikes and spikelets of *P1^POL^* and *P1^WT^* NILs using the same field-grown samples as above. We focused on organ length, given the effects observed in the field (Table 1 andFigure 1D), and all data, including width and area measurements, are presented in Supplemental Data Sets S2 and S3 and Supplemental Figures S4 and S5. The *T. polonicum P1* allele significantly increased glume length (37%; *P *<* *0.001) with respect to the wild-type NILs; this effect was consistent and independent of spikelet position across the spike (Figure 1E). However, we detected a significant gradient in the effect of the *T. polonicum P1* allele across the florets within each spikelet: the largest and most significant effects on lemma length were observed in florets 1 and 2 (28.6% and 19.8%, respectively; *P *<* *0.001), whereas the effect was reduced in floret 3 (+5.8%; *P *<* *0.001) and was nonsignificant in floret 4 (Figure 1F;Supplemental Data Set S3). This gradient in lemma length within spikelets was maintained across all positions along the spike. A very similar gradient within spikelets was also identified for grain length, with *P1^POL^* NILs having significantly longer grains than *P1^WT^* NILs in florets 1 and 2 (+4.1% and +6.7%, respectively; *P *<* *0.001), and nonsignificant differences in grain length in florets 3 (+0.4%; *P *=* *0.37) and 4 (+0.1%; *P *=* *0.92; Figure 1F). Minor effects of the *T. polonicum P1* allele on palea length also followed this spikelet gradient (+2.5% in floret 1 to −3.6% in floret 4; Supplemental Data Set S3 and Supplemental Figure S5). These results suggest that the increases in glume, lemma, and grain length conferred by the *T. polonicum P1* allele are consistent along the spike, but that the positive effects on lemma and grain length follow a basipetal gradient from basal to apical florets within individual spikelets.

To further investigate the differences in grain length between *P1* NILs, we used scanning electron microscopy to image and measure pericarp cell size of *P1^WT^* and *P1^POL^* grains. We selected grains from florets 2 and 4 of central spikelets and imaged the base, center, and distal end of each grain (Supplemental Figure S6A). We found a significant 12.9% increase (*P *<* *0.05) in pericarp cell length in floret 2 grains of *P1^POL^* NILs relative to *P1^WT^* NILs (Figure 1G). This difference in cell length was present only in the central portion of the grain, while cell size was similar between NILs at the base and distal end of the grain (Supplemental Data Set S6 and Supplemental Figure S6). For floret 4, there were no differences in pericarp cell size between the NILs at each of the three positions examined across the grain (Figure 1G;Supplemental Data Set S6 and Supplemental Figure S6). Given the maternal origin of the pericarp, these results are consistent with the *P1^POL^* grain length effect being maternally inherited as first proposed by Engledow (1920). Taken together, these results suggest that the *T. polonicum P1* allele enhances grain size in basal florets through an increase in cell length in the center of the grain, whereas grains of floret 3 and 4 are indistinguishable from the wild-type NILs, both macro- and microscopically.

### *P1* maps to a 50-kb interval on chromosome 7A containing a single candidate gene

**Figure 2:**
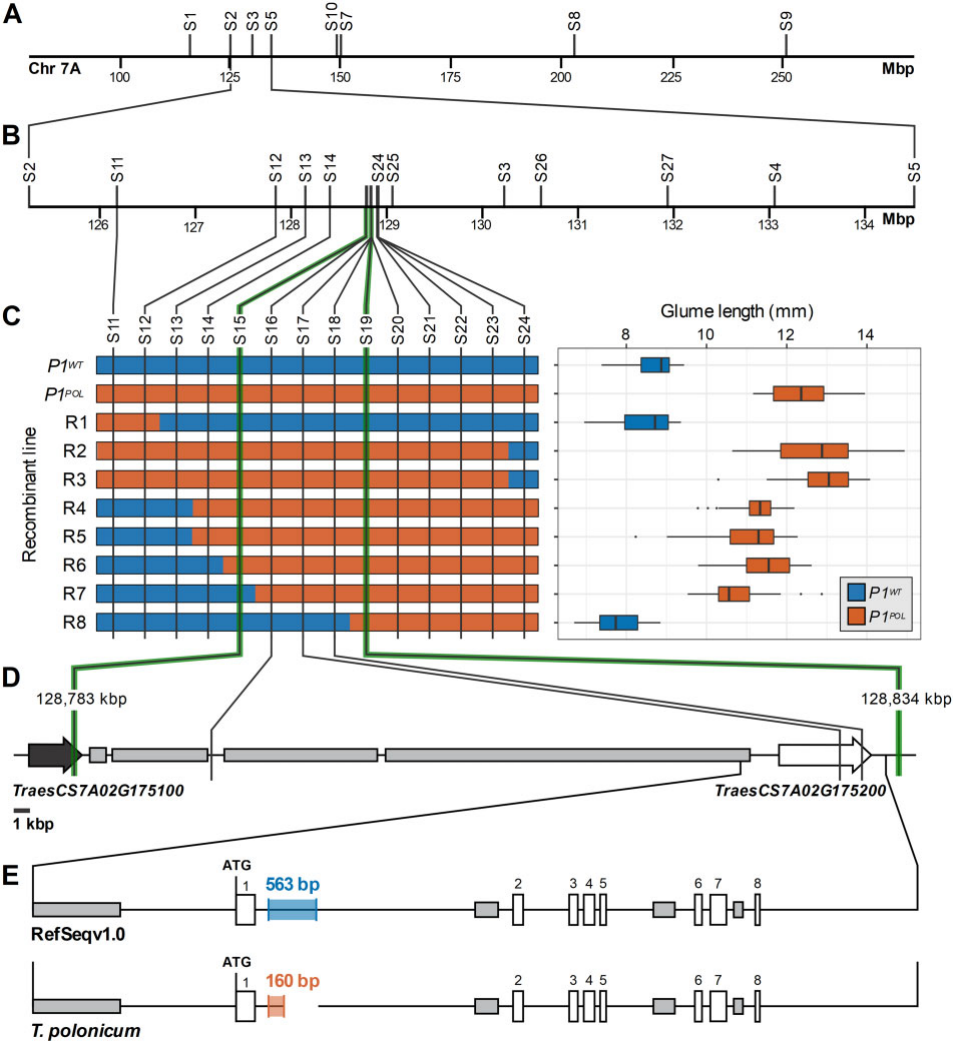
Map-based cloning of the *P1* locus reveals *VRT-A2* as the single candidate gene. A, Initial mapping in 17 BC_4_F_2_ recombinants mapped *P1* between markers *S2* and *S7* (∼25-Mbp interval). B, Subsequently, *P1* was mapped between markers *S2* and *S10* using an additional 64 BC_6_F_2_ recombinant lines. C, Graphical genotype of eight critical recombinants between markers *S12* and *S24* (∼1-Mbp interval; marker distance not drawn to scale). Based on the phenotypic evaluation of glume length, we mapped *P1* to a 50.3-kbp interval between markers *S15* and *S19* (*n*=5 plants per genotype). The box represents the middle 50% of data with the borders of the box representing the 25th and 75th percentile. The horizontal line in the middle of the box represents the median. Whiskers represent the minimum and maximum values, unless a point exceeds 1.5 times the inter-quartile range in which case the whisker represents this value and values beyond this are plotted as single points (outliers). D, The 50.3-kbp interval encompasses the last exon of *Traes7A02G175100* (black arrow), multiple repetitive elements (gray rectangles), and *Traes7A02G175200* (white arrow). E, We identified a single polymorphism between Chinese Spring (RefSeqv1.0) and *T. polonicum* in a ∼10-kbp interval surrounding *Traes7A02G175200*; a 563-bp sequence in RefSeqv1.0 (blue shaded box) was substituted by a 160-bp sequence in *T. polonicum* (orange shaded box)

To map the *P1* locus, we used BC_4_ and BC_6_ recombinant lines derived from the NILs described above. We initially phenotyped 17 BC_4_F_3_ homozygous recombinant lines between markers *S1* and *S9* for glume length and mapped the *P1* locus between markers *S2* (125,260,256 bp) and *S7* (150,240,183 bp; Figure 2A;Supplemental Data Set S7). Heterozygous individuals across the interval had glumes of intermediate length between the homozygous parental lines, consistent with a semi-dominant mode of action of *P1* (Biffen, 1905; Engledow, 1920; Okamoto and Takumi, 2013). To further define the *P1* interval, we screened 1,867 BC_6_F_2_ plants heterozygous across the *S2* and *S7* interval and identified an additional 64 homozygous BC_6_F_2_ recombinants between markers *S2* and *S10*. These recombinants were genotyped with a further 21 markers (Figure 2B;Supplemental Data Set S8). The long-glume phenotype, alongside plant height, spike length, grain length, and thousand grain weight, mapped between markers *S15* and *S19*, spanning a 50,338-bp interval (Figure 2C;Supplemental Data Sets S8 and S9). The complete linkage of the 50.3-kbp region with these multiple phenotypes suggests that they are all pleiotropic effects of the *P1* locus. Note the intermediate glume length phenotype of recombinants R4–R7 relative to R2, R3, and *P1^POL^* (*P *<* *0.01). This could be due to additional genetic elements, proximal to marker *S14* in the *P1^POL^* genetic background, that potentially modulate the magnitude of the *P1* locus.

We identified two gene models based on the RefSeqv1.1 annotation within the *P1* interval: *TraesCS7A02G175100* and *TraesCS7A02G175200*. The flanking marker *S15* resided within the last intron of *TraesCS7A02G175100* and no additional single-nucleotide polymorphisms (SNPs) were detected in the last exon of this gene between *P1^WT^* and *P1^POL^* NILs. Manual annotation of the 50.3-kbp *P1* interval in the RefSeqv1.0 assembly (and an additional 14 hexaploid and tetraploid cultivars; Walkowiak et al., 2020) identified 38,261 bp as repetitive sequences, and no additional gene apart from *TraesCS7A02G175200* (Figure 2D;Supplemental Figure S7A). This suggested *TraesCS7A02G175200* as the sole candidate gene for *P1*.

*TraesCS7A02G175200* encodes a member of the MADS-box gene family previously named *VRT2* in wheat (Kane et al., 2005). *VRT2*, as well as its homolog *TaSVP1*, are the wheat orthologs of *AtSVP* in *A. thaliana* (Supplemental Figure S8; Schilling et al., 2020). Using publicly available RNA-Seq data, we verified the exon–intron structure of *TraesCS7A02G175200.1* (Supplemental Figure S7B). We sequenced the gene in the *P1^POL^* NIL from 2,299 bp upstream of the ATG to marker *S19* (1,857-bp downstream of the termination codon; 9,747 bp total including all exons and introns). Compared to the Chinese Spring RefSeqv1.0 assembly, the only polymorphism was the substitution of a 563-bp sequence in intron-1 for a 160-bp sequence in *P1^POL^* (Figure 2E). We also contrasted the *VRT-A2* sequence from the *P1^POL^* NIL with 18 sequenced wheat accessions (including Paragon—Walkowiak et al., 2020, tetraploid durum—Maccaferri et al., 2019, and wild emmer—Avni et al., 2017) and identified five distinct haplotype groups (Supplemental Data Set S10). The only consistent polymorphism between these haplotypes and the *P1^POL^* sequence was the substitution of the 563-bp intron-1 sequence for the 160-bp sequence. We further analyzed an F_2_ progeny from a cross between Chinese Spring and the *P1^POL^* NIL, which both share the same *VRT-A2* haplotype apart for the intron-1 sequence substitution. The glume length phenotype matched perfectly with the *VRT-A2* allele within these lines (Supplemental Figure S9 and Supplemental Data Set S11), providing additional evidence that the substitution of the 563-bp sequence in intron-1 by the 160-bp sequence is the causal polymorphism. An analysis of this 160-bp sequence suggests that it consists of reoccurring units of DNA that are homologous to sequences flanking the wild-type 563-bp intron-1 sequence (Supplemental Figure S10). These reoccurring sequence units account for 145 of the 160 nucleotides, indicating that the sequence is the result of a local rearrangement rather than an insertion of foreign DNA.

### The 160-bp intron-1 sequence rearrangement in *VRT-A2* is completely linked with the long-glume phenotype in tetraploid and hexaploid wheat accessions

**Figure 3:**
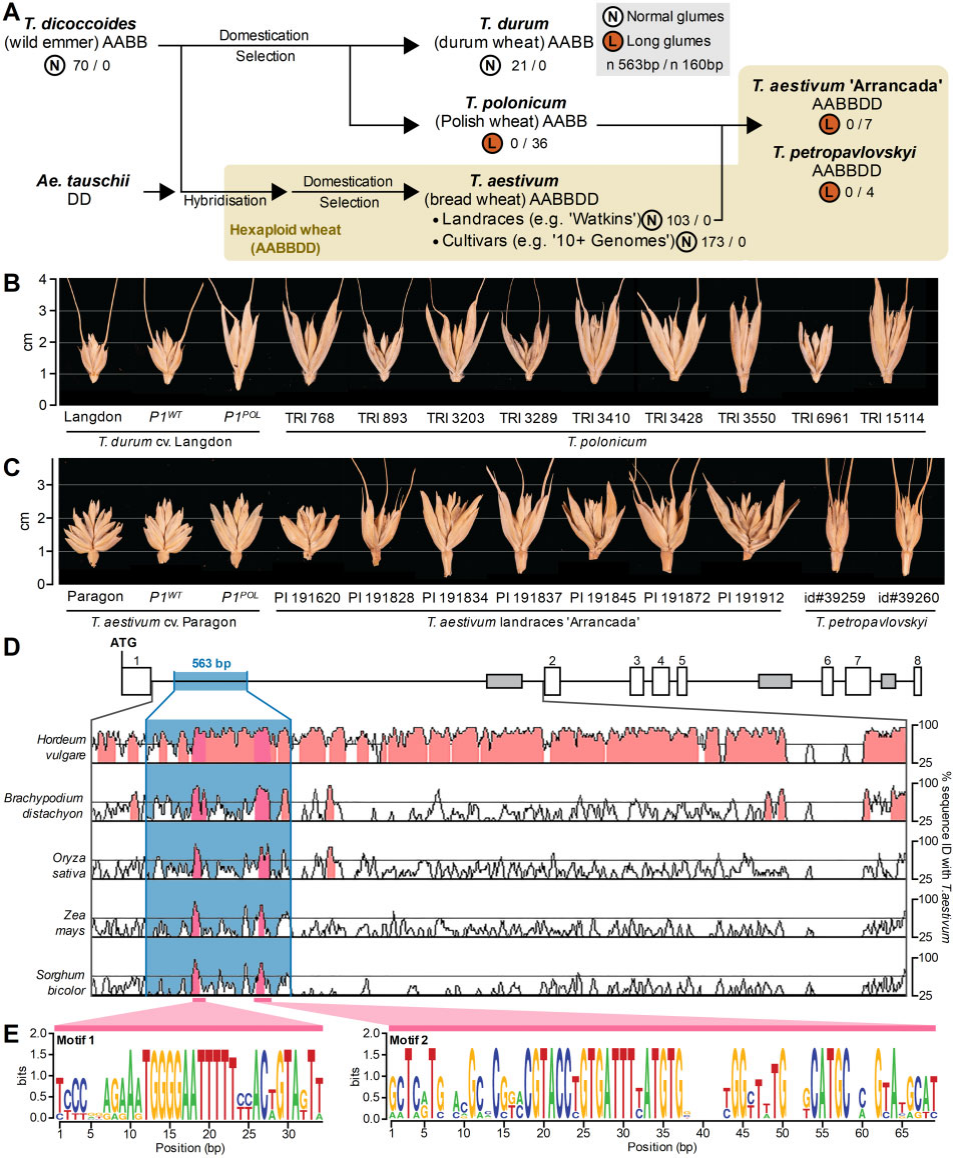
Natural variation of *P1* indicates a single mutation event that led to the loss of evolutionary conserved motifs. A, Simplified diagram depicting the evolution and domestication of tetraploid and hexaploid wheat (tan shaded area). The glume phenotype for each species and set of accessions is indicated by the letter N (normal) or L (long) enclosed in a circle. Beside this classification, the number of accessions that carry the wild-type 563-bp (*VRT-A2a* allele) or the 160-bp (*VRT-A2b* allele) intron-1 sequence is shown. *T. polonicum* hybridized with hexaploid landraces in China and Portugal, giving rise to *T. petropavlovskyi* and the “Arrancada” landrace group, respectively, both of which exhibit long glumes and carry the *VRT-A2b* allele. B, Spikelets of tetraploid wheat including *P1* NILs in the tetraploid cultivar “Langdon” and nine accession of *T. polonicum*. C, Comparison of spikelets of hexaploid wheat including *P1* NILs in the hexaploid cultivar “Paragon”, seven accession from the “Arrancada” landrace group and two accession of *T. petropavlovskyi*. D, Phylogenetic shadowing using mVISTA of *VRT-A2* intron-1 with pairwise alignments of *T. aestivum* with barley (*Hordeum vulgare*), *Brachypodium distachyon*, rice (*O. sativa*), maize (*Z. mays*), and sorghum (*S. bicolor*). The *Y*-axis represents percentage sequence similarity. Two conserved peaks (dark pink) were identified within the 563-bp sequence (blue box) that is absent in *T. polonicum*. E, Sequence of the two conserved motifs that maintain an >80% similarity over a 20-bp sliding window across the species described in (D)

We determined the allelic status of the *VRT-A2* intron-1 sequence rearrangement in a wheat diversity panel. We first screened 367 accessions with wild-type glume length including tetraploid emmer wheat *T. dicoccoides* (*n* = 70), tetraploid durum wheat *T. durum* (*n* = 21), hexaploid wheat landraces from the Watkins collection (*n* = 103), hexaploid UK cultivars (*n* = 98), hexaploid European germplasm from the Gediflux collection (*n* = 60), and 15 sequenced wheat cultivars. We found that all 367 accessions carried the 563-bp sequence in intron-1 and none had the 160-bp sequence rearrangement found in the *P1^POL^* NIL (Supplemental Data Set S12). We thus termed the 563-bp sequence as the wild-type *VRT-A2a* allele, and the 160-bp sequence rearrangement found in *T. polonicum* as the *VRT-A2b* allele, consistent with wheat gene nomenclature guidelines. We next screened 36 accessions of tetraploid *T. polonicum* (all with long glumes) from 17 different countries to determine their *VRT-A2* allele. All 36 *T. polonicum* accessions carried the exact 160-bp rearrangement in intron-1 as the *VRT-A2b* allele (Figure 3, A and B;Supplemental Data Set S13). These results suggest that the *VRT-A2b* allele with its 160-bp sequence rearrangement in intron-1 is unique to *T. polonicum*.

We then examined accessions from two types of hexaploid wheat with long glumes that have been postulated to be the product of independent hybridisation between *T. polonicum* and hexaploid landraces in China (*T. petropavlovskyi*) and Portugal (“Arrancada” group; Figure 3, A and C; Chen et al., 1985, 1988, 2013; Watanabe and Imamura, 2002; Akond and Watanabe, 2005; Akond et al., 2008; Kang et al., 2010). All 11 accessions of *T. petropavlovskyi* (*n* = 4) and the “Arrancada” landraces (*n* = 7) carried the *VRT-A2b* allele found in *T. polonicum* (Supplemental Data Set S13). We fully sequenced the allele (5,591 bp) from six *T. polonicum*, two *T. petropavlovskyi*, and four “Arrancada” accessions (Supplemental Data Set S13) and obtained 100% identical sequences from these 12 long-glume accessions. Using the markers developed for mapping *P1*, we found that the two *T. petropavlovskyi* accessions shared a common haplotype, whereas the seven “Arrancada” accessions also shared a common, albeit distinct, haplotype from that in *T. petropavlovskyi* (Supplemental Data Set S14). Conversely, in accessions with normal-sized glumes, we identified multiple haplotypes within *VRT-A2* (all with the 563-bp intron-1 sequence) and also across the wider physical interval (Supplemental Data Sets S10 and S14). These results, alongside the absence of the 160-bp rearrangement in wild emmer and hexaploid landraces, provide evidence that the 563-bp intron-1 sequence in *VRT-A2a* is ancestral.

### The 563-bp intron-1 sequence of *VRT-A2a* is highly conserved across Poaceae

We compared the entire intron-1 sequence of *VRT-A2a* with orthologous Poaceae sequences from barley (*Hordeum vulgare*), *Brachypodium distachyon*, rice (*Oryza sativa*), maize (*Z. mays*), and sorghum (*S. bicolor*). Phylogenetic shadowing using mVISTA (Mayor et al., 2000; Frazer et al., 2004) revealed two highly conserved regions across Poaceae (>85% sequence id, minimum 20 bp), both of which are missing from the 160-bp rearrangement present in the *VRT-A2b* allele (Figure 3D). We further examined these two regions (see “Materials and Methods”) and identified broadly conserved sequences of 34 and 69 bp in length, hereafter referred to as “Motif 1” and “Motif 2”, respectively (Figure 3E). Within them, both motifs contain highly conserved sequences of 16 and 20 bp, respectively (Supplemental Data Set S15). We searched for putative transcription factor binding sites within Motifs 1 and 2 using three online databases (PlantPan3.0, PlantRegMap, and MEME, see “Materials and Methods”). We found two significant hits (both *P *<* *0.001 and *q *<* *0.001) for Motif 1 encoding members of the LATERAL ORGAN BOUNDARIES-DOMAIN (LBD) family (Supplemental Data Set S16). For Motif 2, we found 17 significant hits (all *P *<* *0.001 and *q *<* *0.05) encoding members of the LBD, Basic Leucine Zipper, B3, and GLABROUS1 enhancer-binding protein families (Supplemental Data Set S16). Given the highly conserved nature of motifs within the 563-bp intron-1 sequence across the investigated Poaceae (∼60 million years divergence time; Charles et al., 2009; Reineke et al., 2011) and the identification of putative transcription factor binding sites, we hypothesize that this intron-1 sequence plays a regulatory role in the expression profile of *VRT-A2*.

### *VRT-A2* is expressed ectopically in *P1^POL^* relative to *P1^WT^* NILs

**Figure 4:**
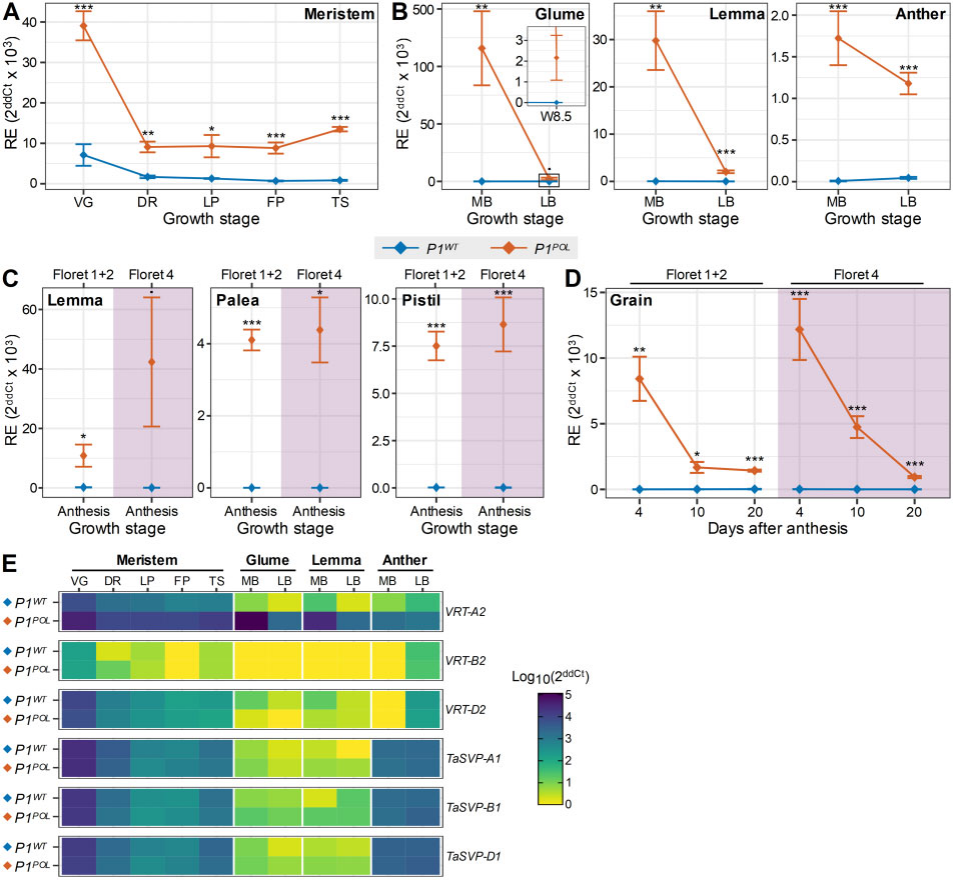
*VRT-A2* is more highly and ectopically expressed in *P1^POL^* relative to *P1^WT^* NILs. A, Relative expression of *VRT-A2* in developing meristems of *P1^WT^* (blue) and *P1^POL^* (orange) NILs. Developmental stages based on Waddington scale (Waddington et al., 1983); VG vegetative meristem (W1); DR, late double ridge stage (W2.5); LP, lemma primordium stage (W3.25); FP, floret primordium stage (W3.5); TS, early terminal spikelet stage (W4). B, Relative expression of *VRT-A2* in glume, lemma (floret 1 + 2), and anther (floret 1 + 2) at mid-boot (MB; W7.5) and late-boot (LB; W8.5) stages. C, Relative expression of *VRT-A2* in lemma, palea, and pistil just before anthesis (W9.5) in florets 1 + 2 (white background) and floret 4 (pink background). Note that for (C) and (D) the growth stage is based on florets 1 + 2; floret 4 tissues will be at a slightly less mature developmental stage. D, Relative expression of *VRT-A2* in grains from florets 1 + 2 as well as floret 4 at 4, 10, and 20 dpa. E, Heatmap showing log_10_ scaled expression in *P1^WT^* and *P1^POL^* NILs for the three *VRT2* and *TaSVP1* homoeologs in tissues and developmental stages shown in (A) and (B) (Supplemental Data Set S17). Values in (A)–(D) are an average 2^ddCt^ ± standard error of the mean from four to five independent biological replicates per tissue*timepoint, run in triplicates. Error bars are mean ± sem. *P < 0.05; **P < 0.01; ***P < 0.001

To assess if the intron-1 sequence rearrangement affected the expression profile of *VRT-A2*, we determined its expression pattern in *P1^WT^* and *P1^POL^* NILs using reverse-transcription quantitative polymerase chain reaction (RT-qPCR). We first examined expression levels in developing meristems of *P1^WT^* NILs. Consistent with previous studies (Kane et al., 2005, 2007; Trevaskis et al., 2007), we found a progressive decrease in *VRT-A2* expression from vegetative meristem (W1) to terminal spikelet stage (W4; Figure 4A). We next examined expression in *P1^POL^* NILs, which showed a 5- to 16-fold higher expression level of *VRT-A2* relative to the *P1^WT^* NILs at all five timepoints investigated (*P *<* *0.05; Figure 4A). An increased expression level of *VRT-A2* in *P1^POL^* relative to *P1^WT^* was also observed in leaves at the same developmental stages (6- to 38-fold higher, *P *<* *0.05; Supplemental Data Set S17).

Next, we examined *VRT-A2* expression in developing glumes, lemmas, anthers, flag leaves, and grains at multiple developmental timepoints. In the wild-type NIL, expression was restricted to the flag leaves and anthers at anthesis (Figure 4B;Supplemental Data Set S17). We did not detect *VRT-A2* expression in glumes, lemmas, nor grains of *P1^WT^* NILs at any timepoint (Figure 4, B–D), consistent with publicly available RNA-Seq data of wild-type *VRT-A2* genotypes (Borrill et al., 2016; Ramirez-Gonzalez et al., 2018). In contrast, *VRT-A2* expression was detected in all tissues and at all timepoints in the *P1^POL^* NIL, including glumes, lemmas, and grains (Figure 4, B–D).

Given the contrasting effects of the *VRT-A2b* allele on grain and lemma length between florets 1 + 2 and 4 (Figure 1F), we compared the *VRT-A2* ectopic expression in these samples. Across multiple tissues and timepoints (lemma, palea, pistil at anthesis; grains at 4, 10, and 20 dpa), we found similar expression of *VRT-A2* in samples from florets 1 + 2 compared to floret 4 (Figure 4, C and D). We also investigated expression of the *VRT2* homoeologs (*TraesCS7B02G080300*, *TraesCS7D02G176700*), and that of the closely related ortholog *TaSVP1* (*TraesCS6A02G313800*, *TraesCS6B02G343900*, *TraesCS6D02G293200*; Supplemental Figure S8; Schilling et al., 2020). Across the same tissues and developmental timepoints as those described above, we did not detect any differences in expression between *P1^WT^* and *P1^POL^* NILs (Figure 4E). These results show that *VRT-A2* is expressed more highly and ectopically in *P1^POL^* relative to *P1^WT^* NILs across multiple tissues and timepoints. The ectopic expression does not extend to the homoeologs or closely related orthologs and is not restricted to those tissues in which we observed phenotypic differences between *P1^WT^* and *P1^POL^* NILs.

### Ectopic expression of *VRT-A2* leads to phenotypic effects in a dosage-dependent manner

We next tested whether the observed changes in *VRT-A2* expression patterns in *P1^POL^* NILs are causal for the *T. polonicum* long-glume phenotype. We transformed the hexaploid accession “Fielder” (normal glume phenotype) using the genomic *T. polonicum VRT-A2b* allele (5,591 bp), including 2,299-bp upstream of the ATG, all coding and intron sequences, as well as 1,000-bp downstream of the termination codon (construct pGGG-AH-VRT-A2; Supplemental Figure S11). We obtained 14 independent T_0_ lines, which were classified based on their transgene copy number. No transgene was detected for five lines (zero copy number), three lines carried 1 or 2 copies (low copy number), three lines carried 4 to 5 copies (medium copy number), and three lines carried 9–35 copies (high copy number; Supplemental Figure S12 and Supplemental Data Set S18–S20).

We collected tissue from flag leaves, glumes, and grains (florets 1 and 2) at 21 dpa from all 14 T_0_ plants to measure expression levels of *VRT2* homoeologs (Supplemental Data Set S18). We detected expression of *VRT-A2* in flag leaves of all 14 transformed lines, including the zero copy number lines, similar to that observed in *P1^WT^*. In glume and grain tissue, *VRT-A2* expression was extremely low or not detected in the zero copy number lines, consistent with the *P1^WT^* NILs (Figure 4), whereas we detected expression in all lines with at least one copy of the transgene. In all tissues, *VRT-A2* expression scaled with copy number. As seen in the NILs, we did not detect differences in expression of the B- and D-homoeologs among transgenic lines (Supplemental Data Set S18). We then selected two independent events each for the zero, low, medium, and high copy number classes and grew five T_1_ plants for event. We collected tissue from flag leaves, glumes, grains (floret 2), and rachis at 14 dpa for all 40 T_1_ plants. Consistent with the T_0_ generation, we detected *VRT-A2* expression in flag leaves from all plants. In glumes, grains, and rachis, *VRT-A2* expression was detected only in the low, medium, and high copy number lines, whereas expression was undetectable or extremely low in zero copy lines (Supplemental Data Set S18).

**Figure 5:**
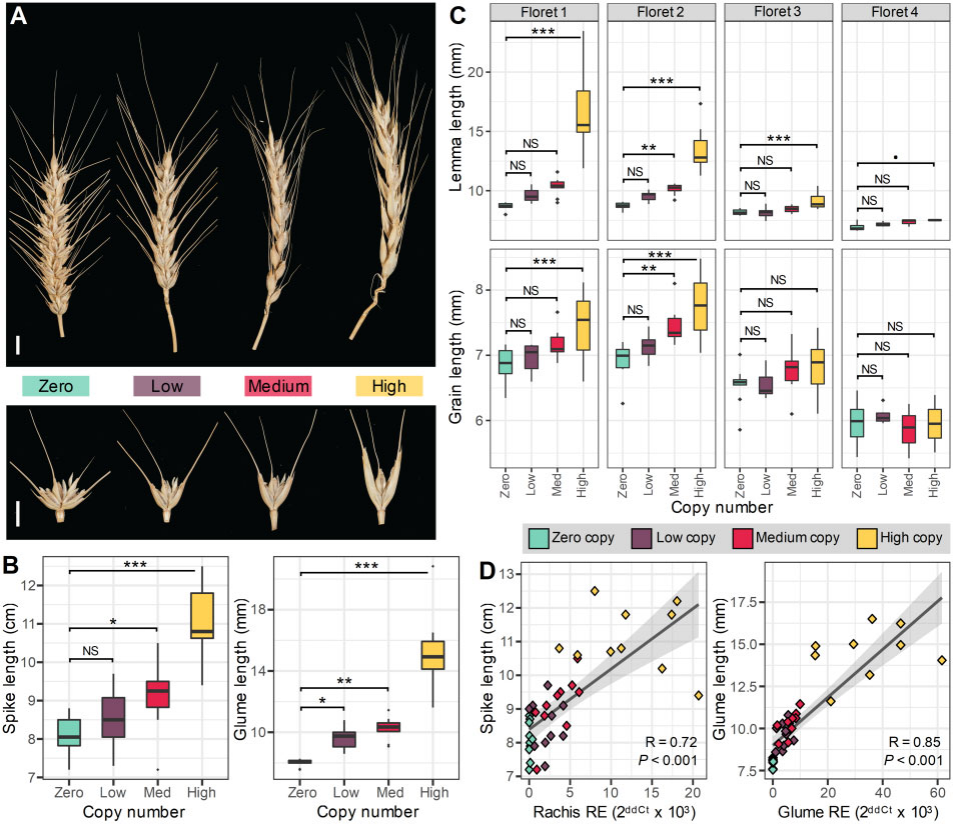
Ectopic *VRT-A2* expression elicits phenotypic effects in a dosage-dependent manner. A, Comparison of spikes and spikelets of zero, low (1–2), medium (3–4), and high (9–35) copy number lines (left to right). Notably, spike length increases with copy number, as does glume length. Scale bar = 1 cm. B, Box plots depicting the variation of spike (left) and glume (right) length, respectively, from tillers of zero (cyan), low (purple), medium (red), and high (yellow) copy number lines (*n*=10 plants per boxplot). Horizontal lines represent the median. C, Box plots depicting lemma and grain length for florets 1, 2, 3, and 4 for zero (cyan), low (purple), medium (red), and high (yellow) copy number lines (*n*=10 plants per boxplot). D, Pearson correlations between *VRT-A2* relative expression in the rachis at 14 dpa and spike length (left, *n* = 40 plants), and in the glume at 14 dpa and glume length (right, *n* = 39 plants). Relative expression shown as 2^ddCt^ × 10^3^ (Supplemental Data Set S18). Regression (Dark gray line) and 95% confidence interval (light gray shading) are shown. Data points are colored according to copy number. Additional correlations in Supplemental Data Set S20. Note that for the glume length correlation we excluded one value as the both the relative expression and glume length were outlier values. The correlation plot including this outlier value is presented as Supplemental Figure 13. The box represents the middle 50% of data with the borders of the box representing the 25th and 75th percentile. The horizontal line in the middle of the box represents the median. Whiskers represent the minimum and maximum values, unless a point exceeds 1.5 times the interquartile range in which case the whisker represents this value and values beyond this are plotted as single points (outliers). Statistical classifications in (B) and (C) are based on Dunnett tests against the zero copy number lines. *P* < 0.10; *P < 0.05; **P < 0.01; ***P < 0.001

We measured the length of the main spike for each of the 40 T_1_ plants, before dissecting all spikelets from the main spike for morphological characterization. We compared spike and glume length, as well as lemma, palea, and grain length from florets 1 to 4 among the four categories of copy number lines (Figure 5, A–C; Supplemental Data Set S19). Overall, we identified significant differences between the zero copy lines (*N* = 10) and the transgenic lines (low, medium, and high; *N* = 30) for spike, glume, lemma, palea length (all *P *<* *0.004), and grain length (*P *=* *0.01; Supplemental Data Set S19). These differences were largest for glume (46%), lemma (27%), and spike length (19%), whereas palea and grain length showed more discrete, albeit significant, effects (6% and 5%, respectively; Figure 5, B and C;Supplemental Data Set S19). These results were almost identical to those obtained from the T_0_ generation (Supplemental Data Set S19).

Having established the overall positive effects of the *T. polonicum VRT-A2* transgene on these traits, we next evaluated the magnitude of the phenotypic effects among the four categories of copy number lines. Glume length significantly increased with copy number from low (20%; *P *=* *0.03), to medium (28%; *P *=* *0.002), and to high (89%; *P *<* *0.0001) copy number lines relative to the zero copy number lines (Figure 5B;Supplemental Data Set S19). We further detected significant effects in the medium (all *P *<* *0.03) and high copy number lines (all *P *<* *0.001) for lemma length (15% and 59%, respectively), grain length (5.5% and 8.6%, respectively), and palea length (5.5% and 12%, respectively) in comparison to the zero copy number lines (Supplemental Data Set S19). The low copy number lines showed a nonsignificant increase in lemma length (6.1%; *P *=* *0.48) but were almost identical to the zero copy number lines for grain and palea length (Supplemental Data Set S19). Similar to lemma length, we observed a nonsignificant increase in spike length in the low copy number lines (5.7%; *P *=* *0.43), while the medium (13%; *P *=* *0.02) and high copy number lines (37%; *P *<* *0.0001) had significantly longer spikes (Figure 5B;Supplemental Data Set S19). These results were consistent with those observed in the T_0_ generation (Supplemental Data Set S19).

We observed a gradient in the phenotypic effects from florets 1 to 4, with the basal florets showing the strongest effect, while more apical florets showed reduced or no effects (Figure 5C;Supplemental Data Set S19). This is consistent with what we observed in the *P1* NILs. This basipetal gradient was most obvious in the high copy number lines. For example, in floret 1 of the high copy number lines, lemma, palea, and grain length increased by 90%, 14%, and 8.4%, respectively, whereas in floret 3 those tissues only increased by 11%, 5.8%, and 4.3%, respectively (*P *<* *0.008, except grain length in floret 3, *P *=* *0.13; Figure 5C;Supplemental Data Set S19). This same gradient was also present in the T_0_ generation.

To further document the increased magnitude of the phenotypic response with increasing levels of *VRT-A2* copy number, we analyzed the actual expression of *VRT-A2* in different organs to relate it to the phenotypic data in the different transgenic lines. *VRT-A2* expression correlated highly and significantly with several phenotypic traits, independent of the tissue examined for expression. For example, *VRT-A2* expression levels in the rachis correlated highly with spike length (*R*^2^ = 0.52; *P *<* *0.0001), while expression in glumes correlated strongly with glume length (*R*^2^ = 0.78; *P *<* *0.0001; (Figure 5D), as well as with lemma, palea, and grain length in floret 1 (*R*^2^ = 0.80, 0.58, and 0.49, respectively; all *P *<* *0.0001; Supplemental Data Set S20). Similar to the gradient in phenotypic effects across florets detailed above, these correlations were strongest and most significant in florets 1 and 2 (*R*^2^ > 0.46), remained significant for floret 3 lemma and grain phenotypes (*R*^2^ ≥ 0.16), and were nonsignificant for floret 4 phenotypes (Supplemental Data Set S20). The multiple phenotypes of the low, medium, and high copy transgenic lines recreate, in a dosage-dependent manner, the effects seen in the *P1^POL^* NIL, providing further evidence that *VRT-A2* is the causal gene underlying the *T. polonicum P1* locus.

## Discussion

### An intron-1 sequence rearrangement in *VRT-A2* defines the subspecies *T. turgidum* ssp. *polonicum*

The first formal classification of wheat was compiled by Linnaeus in 1,753 and was based on discernible characteristics such as phenology, spike architecture, and glume morphology. With its characteristic long glumes, *T. polonicum* is a standout *Triticum* subspecies. Despite this, the first mention of *T. polonicum* only dates back to 1687 (Percival, 1921). *Triticum polonicum* was instrumental in the study of early geneticists who showed that measurable quantitative traits, such as glume length, were also inherited according to the same laws of qualitative traits postulated by Mendel (Biffen, 1905; Engledow, 1920). However, despite first being described genetically over a century ago, the gene underlying the *P1* locus remained unknown.

Here, we show that the gene underlying the long-glume *P1* locus of *T. polonicum* is *VRT-A2*, which encodes a member of the SVP family of MADS-box transcription factors. We mapped multiple phenotypes associated with *P1*, including glume length, grain length, spike length, grain weight, and plant height to the same physical interval that included *VRT-A2* as the single candidate gene. For grains, we further showed that the increase in length in *P1^POL^* is likely the result of increased cell length in the central portion of the grain, but not at the base or distal end. While we detected ectopic *VRT-A2* expression at several timepoints during grain development, our approach lacked the necessary spatial resolution to determine whether this uneven response in the grain is due to localized expression of *VRT-A2* or the result of external factors, e.g. an increased volume of the floral cavity of the *P1^POL^* NIL allowing the grain to extend further than in the wild-type (Millet, 1986).

We identified a sequence rearrangement in the first intron of *VRT-A2* in which a 563-bp wild-type sequence (*VRT-A2a*) was replaced by a 160-bp fragment in *T. polonicum* (*VRT-A2b*). The 160-bp sequence is mostly composed of imperfect tandem copies of two sequence units that flank the rearranged intron-1 region (Supplemental Figure S10). The 160-bp sequence has no match to other plant sequences or to repetitive elements consistent with its local origin rather than an insertion of foreign sequence. We hypothesize that the rearrangement was caused by erroneous DNA repair following a double-strand break via the alternative nonhomologous end-joining (aNHEJ) pathway (Deriano and Roth, 2013; Rodgers and McVey, 2016). aNHEJ has been shown to result in complex insertion/deletion events with insertions being identical or near-identical matches of flanking sequences (Kent et al., 2016; van Kregten et al., 2016). These templated insertions are associated with many disease-causing genomic rearrangements in humans (Schimmel et al., 2019).

Using diverse germplasm, we showed that the *VRT-A2b* allele is only present in tetraploid *T. polonicum* accessions, or in hexaploid wheat germplasm with long glumes. Our results provide strong evidence that these hexaploid wheat accessions with long glumes, namely *T. petropavlovskyi* and the “Arrancada” accessions, are the outcome of hybridizations between *T. polonicum* and hexaploid wheat (Chen et al., 1985, 1988, 2013; Watanabe and Imamura, 2002; Akond and Watanabe, 2005; Akond et al., 2008; Kang et al., 2010). The lack of sequence variation among lines with the *VRT-A2b* allele, coupled with its absence among ancestral wheat types (e.g. wild emmer, Watkins landraces), suggests a single and recent origin for the *VRT-A2b* allele. We propose that a single mutation event (possibly through aNHEJ) in the ancestral *VRT-A2a* intron-1 sequence gave rise to the 160-bp sequence rearrangement within the domesticated tetraploid gene pool. This mutation was later introduced into hexaploid wheat via hybridization resulting in hexaploid accessions with long glumes. These results, together with the complete linkage of this *VRT-A2b* allele with the long-glume phenotype, suggest that the intron-1 mutation in *VRT-A2b* is the polymorphism defining the subspecies *T. turgidum* ssp. *polonicum*.

### *VRT-A2* expression levels affect spike, glume, grain, and floral organ length in a dosage-dependent manner

We observed that *VRT-A2* was expressed to a higher degree in *P1^POL^* compared to *P1^WT^* NILs in all tested tissues (e.g. developing spikelets, leaves, and anthers). We also observed ectopic *VRT-A2* expression in *P1^POL^* tissues that have no detectable expression in wild-type lines (e.g. glumes and grains). It is important to note, however, that increased and ectopic expressions are not completely independent categorizations. The resolution afforded by our RT-qPCR analysis is insufficient to discern between higher absolute expression of *VRT-A2b* or the possibility of the gene being ectopically expressed in additional regions of the tissue being sampled. This latter scenario would manifest as “higher” relative expression in the RT-qPCR, when in fact it should be interpreted as ectopic expression. Further in situ experiments will be required to distinguish between these possibilities. The ectopic expression profiles seen in *P1^POL^* were also found in the two generations of transgenic lines carrying the *VRT-A2b* allele. In these transgenic lines, we found significant positive correlations between *VRT-A2* expression levels and the magnitude of the phenotypic effects on glume, lemma, palea, grain, and spike length (Figure 5D;Supplemental Data Set S20). Equivalent dosage relationships were seen in *VRT-A2* overexpression lines for glume length (Li et al., 2020). These results suggest that ectopic expression of *VRT-A2* leads to multiple phenotypic effects, including elongated spikes, glumes, grains, and floral organs, in a dosage-dependent manner in polyploid wheat.

We observed two consistent phenotypic gradients due to the misexpression of *VRT-A2*, the first along the spikelet and the second within the individual florets. In the first gradient, the NILs and transgenic plants show the strongest phenotypic effects in the glumes and basal florets of the spikelets (florets 1 and 2), and the magnitude of these effects lessens in more apical sections of the spikelet (florets 3 and 4), consistent with a basipetal gradient that determines organ length. In the second gradient, outer organs, including glumes and lemmas, are more responsive to changes in *VRT-A2* expression levels than inner organs of the floret such as paleae and carpels. In the transgenic lines, both of these gradients respond to, and scale, with *VRT-A2* expression levels in a dosage-dependent manner. Thus, the strongest phenotypic effects in transgenic lines are observed in glumes and lemmas of basal florets of high copy number lines, while the most muted effects are found in the paleae and grains of florets in the distal spikelet positions in low copy number lines.

A possible explanation for these results is the sequential formation of these organs during spikelet and floral development together with the fact that MADS-box genes act in a sequential manner as part of protein complexes (Schwarz-Sommer et al., 1992; Goto and Meyerowitz, 1994; Davies et al., 1996; Huang et al., 1996; Riechmann et al., 1996; Egea-Cortines et al., 1999; Honma and Goto, 2001). In the wild-type, *VRT2* expression is strongly downregulated during the transition from vegetative meristem to the double ridge stage, presumably to allow floral transition from vegetative to spikelet and floret meristems (Trevaskis et al., 2007; Li et al., 2019, 2020). Tetraploid wheat lines with constitutive expression of *VRT2* show significant downregulation of A-, B-, C-, and E-class floral genes at the terminal spikelet stage of spike development (W3.5; Li et al., 2020). In Arabidopsis, the SVP-class genes *SVP* and *AGL24* act as repressors of B- and C-class flowering genes (Gregis et al., 2013). Thus, normal transition into floral meristems takes place under decreasing SVP levels and increasing levels of A-class (SQUAMOSA) and E-class (SEPALLATA) MADS-box proteins, among others.

Disruption of this balance can lead to increased vegetative characteristics as evidenced in E-class mutants in rice that have leaf-like glumes (rudimentary glumes and sterile lemmas), lemmas, and paleae (Ren et al., 2016; Wu et al., 2018). Likewise, overexpression of *SVP* genes in wheat (Li et al., 2020) and barley (Trevaskis et al., 2007) results in a delay or reversion, respectively, of this vegetative to reproductive transition. Li et al. (2020) hypothesize that the downregulation of *SVP* genes is necessary given that SVP proteins interfere with SQUAMOSA–SEPALLATA protein complexes that are required for normal spikelet and floral development. This is consistent with the results presented here where ectopic expression of *VRT-A2* and its prolonged presence in *P1^POL^* and transgenic lines would interfere with the activity/assembly of these protein complexes. The magnitude of the response and the tissues affected would be dependent on the level of *VRT2* expression and the ability of VRT2 proteins to compete with the sequential MADS-box protein complexes that give rise to the different floral tissue types. We hypothesize that VRT2 is able to compete more strongly with protein complexes required for glume and lemma development (as shown in Li et al., 2020) and gradually less so with those protein complexes involved in palea development, which include additional MADS-box proteins (e.g. *AGL6-like* genes; Reinheimer and Kellogg, 2009). Likewise, the increasing levels of A-class and E-class MADS-box proteins during spikelet development would gradually “out-compete” the presence of ectopic VRT2 and thus lessen its phenotypic effects in the more distal, and later produced, florets. This would explain the dosage-dependent response observed in our study and why we observe the strongest effects in outer and early established organs (e.g. glumes and lemmas) of basal florets while later developing/differentiating organs (e.g. paleae, florets 3 and 4) are affected only in lines with the highest *VRT-A2* expression.

### Intron-1 motifs are conserved across grasses and may be recognized by repressors

Our results are reminiscent of the pod corn phenotype observed in maize *Tunicate1* (*Tu1*) mutants, in which the grains (kernels) are completely enclosed by elongated glumes. Similar to *P1*, the mutant *Tu1* phenotype is caused by the ectopic expression of the MADS-box gene *ZMM19*, the maize *TaSVP1* homolog, and a closely related ortholog of wheat *VRT2*, in the developing maize inflorescence (Supplemental Figure S8; Han et al., 2012; Wingen et al., 2012; Schilling et al., 2020). The ectopic expression of *ZMM19*, however, is due to a duplication and rearrangement in the promoter region, whereas our results indicate that the intron-1 sequence plays a key regulatory role in the expression profile of *VRT-A2*.

Numerous MADS-box genes have been shown to contain regulatory sequences within their first introns, including *FLOWERING LOCUS C* in Arabidopsis (Sung et al., 2006) and *VRN1* in wheat (reviewed in Distelfeld et al. (2009)). We thus hypothesize that the 563-bp sequence of the *VRT-A2a* allele, substituted for 160-bp in the *VRT-A2b* allele, contains putative regulatory sequences for establishing the correct expression pattern of the gene. By comparing *VRT2* intron-1 sequences across Poaceae, we identified two distinct motifs (both within the 563-bp region of intron-1) that showed a high degree of sequence conservation across 60 million years of evolution. The absence of the 563-bp intron-1 sequence, as in *VRT-A2b*, results in the spatiotemporal misexpression of *VRT-A2*. It is thus tempting to speculate that either one or both conserved intron-1 motifs allows the binding of proteins or protein complexes that repress *VRT-A2* expression. Currently though, we cannot exclude the possibility that the misexpression of *VRT-A2* is caused by the 160-bp sequence substitution of the *VRT-A2b* allele. Despite the 160-bp sequence not being homologous to any plant sequence in the National Center for Biotechnology Information (NCBI) database, it is predicted to contain putative DNA binding motifs for transcription factors (Supplemental Data Sets S15 and S16). In future work, CRISPR-Cas9 induced deletions in a wild-type background should allow us to (1) confirm that the deletion of the 563-bp intron-1 sequence is sufficient to recapitulate the long-glume *P1* phenotype and (2) define the minimal sequence variation required for ectopic expression, for example, by targeting each intron-1 motif separately and in combination. These different deletions lines, combined with more in-depth spatial and temporal expression analyses (e.g. in situ hybridization or spatial transcriptomics; Giacomello et al., 2017), will provide even greater clues as to the role of this intron-1 sequence in regulating *VRT2* expression. It will also be exciting to investigate if CRISPR-Cas9 induced deletions of the corresponding motifs in other Poaceae results in ectopic expression of *SVP* orthologs and analogous floral organ phenotypes.

Using online databases, we identified significant hits in both intron-1 motifs to members of the LOB-domain family of transcription factors. LOB-domain genes are important for the establishment of boundaries between floral organs and have been shown to be important for glume, lemma, and palea development in several monocot species. In rice, mutations in LOB-domain genes *DEGENERATED HULL 1* (*DH1*; Li et al., 2008) and *INDETERMINATE GAMETOPHYTE 1* (*OsIG1*; Zhang et al., 2015) affect glume, lemma, and palea formation, and *OsIG1* affects expression of *SEPALLATA* and *AGL-6* like MADS-box genes. In maize and barley, LOB-domain genes *RAMOSA2 (RA2*; Bortiri et al., 2006) and *VULGARE ROW-TYPE SPIKE 4* (*VRS4*; Koppolu et al., 2013) restrict inflorescence branching and establish determinacy of spikelet meristems. Strong overexpression of *VRT-A2* in wheat (Li et al., 2020), and maize plants with multiple copies of the ectopically expressed *Tu1* allele (Han et al., 2012), result in spikelet branching similar to that observed in *ra2* mutants (Bortiri et al., 2006). This is consistent with an antagonistic relationship between their activities as first suggested by Han et al. (2012). Further investigation, for example through yeast-one-hybrid, will be required to understand if the intron-1 motifs can be recognized by repressors and if LOB-domain proteins play a role in this.

The B- and D-homoeologs of *VRT2* also contain the two highly conserved intron-1 motifs, and as such we see no difference in their expression pattern in NILs or in transgenic lines. Likewise, no changes in expression of the closest MADS-box ortholog (*TaSVP1*) were detected in *P1* NILs, similar to the lack of expression differences in closely related MADS-box genes in the maize *Tu1* mutants (Han et al., 2012; Wingen et al., 2012). This suggests that *VRT-A2* either does not regulate its homoeologs or is unable to overcome the presence of the putative repressive protein or protein complex in intron-1 of the B- and D-genome homoeologs. Further work, as described above, will be required to fully characterize the role of these putative motifs and how they regulate expression of *VRT2*.

### cis-regulatory variation can impact agronomic traits in polyploid wheat

Major loci that control a relatively large proportion of phenotypic variation for quantitative traits have been selected during domestication of diploid plant species (reviewed in Swinnen et al. (2016)). Often, the causal variants underlying these phenotypes occur in cis-regulatory regions of developmental regulators that affect the level or the spatiotemporal expression profile of transcription factors (Sieburth and Meyerowitz, 1997; Salvi et al., 2007; Louwers et al., 2009; Studer et al., 2011). Selection of cis-regulatory variation has also played a pivotal role in shaping polyploid wheat domestication. Examples include the major vernalization (*VRN1*; Yan et al., 2003) and photoperiod (*Ppd1*; Wilhelm et al., 2009) response genes as well as the major homoeolog pairing *Ph1* locus (Rey et al., 2017). All these selected wheat domestication alleles are dominant or semi-dominant, thereby circumventing functional redundancy and allowing the rapid detection of favorable phenotypes.

The *P1^POL^* allele provides a compelling example, where the extended expression of *VRT-A2* results in enhancement of traits of agronomic interest in a dosage-dependent (semi-dominant) manner. This is similar to recent results in maize, where increasing and extending the expression of the MADS-box gene *ZMM28* resulted in improved vegetative and reproductive growth parameters, which impacted positively on yield (Wu et al., 2019). Interestingly, the authors discuss how a more subtle over- and extended expression of *ZMM28* using a native maize promoter resulted in more consistent yield benefits and fewer pleiotropic effects compared to promoters with constitutive overexpression. Analogously, the overexpression of *VRT-A2* in wheat (Li et al., 2020) and related *SVP* genes in barley (Trevaskis et al., 2007) and rice (Sentoku et al., 2005) using the maize Ubiquitin promoter (in all three studies) resulted in multiple negative pleiotropic effects, including floral reversion. These results highlight how the more subtle changes in expression profiles, through variation in cis-regulation, can impact on agronomic traits. Recent work in tomato has shown how a wide range of phenotypic variation for quantitative traits can be engineered by genome editing of transcription factor promoters to generate cis-regulatory alleles (Rodriguez-Leal et al., 2017). It will be important to determine if engineered cis-regulatory variants will overcome functional redundancy and have similar impact on agronomic traits in a polyploid context.

In summary, we identified *VRT-A2*, a member of the *SVP* family of MADS-box transcription factors, as the gene underlying the *T. polonicum P1* locus in polyploid wheat. An intron-1 sequence rearrangement results in the misexpression of *VRT-A2*, which leads to multiple phenotypic effects in a dosage-dependent manner. Allelic variation studies support the intron-1 mutation in *VRT-A2* as the polymorphism defining the *T. polonicum* subspecies. The *VRT-A2b* allele increases grain weight and other agronomic traits, but not yield, in UK environments. As expression levels of *VRT-A2* are correlated with the magnitude of the phenotypic effects, it is possible that engineering of *VRT2* expression patterns through novel cis-regulatory alleles will generate further beneficial quantitative variation for plant breeding.

## Materials and methods

### Germplasm

To develop the *P1* NILs, we crossed *T. polonicum* accession 1100002 to the hexaploid spring wheat (*T. aestivum*) cultivar Paragon and the resulting F_1_ was backcrossed four to six times to the Paragon recurrent parent. At each generation, F_1_ lines exhibiting the long-glume phenotype of *T. polonicum* where selected to continue the backcrossing process. After four (BC_4_) or six (BC_6_) backcrosses, BC_n_F_2_ plants were grown and homozygous lines for *P1* selected based on glume length. Bulked seed from the BC_4_F_2_ or BC_6_F_2_ plants were used for subsequent experiments. Accessions of *T. dicoccoides*, *T. polonicum*, *T. petropavlovskyi*, and of the *T. aestivum* landrace group “Arrancada” as well as the Watkins collection were obtained from the IPK Genebank, the USDA-ARS National Small Grains Collection, the John Innes Centre Germplasm Resources Unit, the Centre for Genetic Resources at Wageningen University, and the International Center for Agricultural Research in the Dry Areas.

### Field experiments and phenotyping

The *P1* NILs were evaluated in six field experiments between 2016 and 2020. Two trials (2016 BC_4_; 2020 BC_4_ and BC_6_) were sown at the John Innes Centre Experimental trials site in Bawburgh, UK (52°37'50.7"N 1°10'39.7"E) and four (2017 BC_4_; 2018, 2019 and 2020 BC_4_ and BC_6_) were sown at The Morley Agricultural Foundation trials site in Morley St Botolph, UK (52°33'15.1"N 1°01'59.2"E). All experiments were sown in autumn (end September–November; except 2020 which was sown in February) as yield-scale plots (6m × 1.2m) in a randomized complete block design with five replications and sown by grain number for comparable plant densities aiming for 275 seeds·m^−2^. Developmental traits were evaluated throughout the growing period and a 10-ear sample was collected at harvest for the assessment of spike, floret, and grain characteristics (marked “10ES” in Supplemental Data Set S1). Spike length was measured as the distance between the peduncle–rachis junction and tip of the terminal floret. Plot yield, hectoliter weight, and grain moisture were measured during harvest on board the combine (Zürn 150). Final grain yield was determined per plot after adjustment to 15% grain moisture. Grain morphometric measurements were analyzed using the MARVIN grain analyzer (GTA Sensorik GmbH, Neubrandenburg, Germany) using approximately 400 grains of the combined grain samples.

### Spike dissection and organ measurements (NILs and transgenic lines)

We measured organ size of the *P1^WT^* and *P1^POL^* NILs by sampling three spikes from five field blocks per NIL grown in 2019 at Morley. The spikes were dissected by hand and all organs (glume, lemma, palea, and grain) were placed on PCR film (Cat No.: AB0580, Thermofisher) from bottom (position 0 in Figure 1, E–F) to top (position 20 in Figure 1, E and F) of the spike. The PCR films with the organs were scanned using a standard Ricoh photocopier (settings: greyscale, 600 dpi). The resulting images were analyzed using the Fiji “analyze particles” function, restricting analysis to particles of 0.1–5 cm^2^ area (Schindelin et al., 2012). Fiji measures particles from top-left to bottom-right of the image, thus allowing us to match position of the organ along the spike with the Fiji measurements retrospectively. To phenotype the Chinese Spring – *P1^POL^* population, we collected six glumes from three central spikelets of the main spike for 168 F_2_ plants and measured their length as described above. To measure organ size in the transgenic T_0_ and T_1_ lines (grown in 1 L pots under 16 h light at 20°C and 8 h darkness at 15°C in a controlled environment room), we hand dissected organs from a single (T_1_) or two (T_0_) main spikes per plant. For the T_0_ transgenics we used five zero copy number plants, three low copy plants, three medium copy plants, and three high copy number plants. For the T_1_ transgenics, we used five plants for eight events (two zero, two low, two medium, and two high copy number events). The organs were measured and analyzed as described for the NILs above.

### 3D scanning of spikes and morphometric grain extraction

Fifteen mature spikes from both *P1* NILs grown in 2019 at Morley were used for μCT scanning (three spikes from five field blocks per NIL). Scanning conditions were as described in Hughes et al. (2019). Feature extraction from the scans was performed using previously developed MATLAB‐based software (Hughes et al., 2017) using the following setup parameters (se = 7, voxel size = 68.8, minSize = 10,000 and watershed=false). The features extracted were length (calculated using the major axis of the whole grain), width, and depth (the major and minor axis of a cross-section, respectively, found by selecting the grain’s midpoint), volume (a complete connected pixel count per grain), and grain counts for each spike. More than 750 grains were measured per genotype. The data were checked for false positives by first removing outliers that were identified using the 0.025 upper and lower percentiles of the data. Additionally, for added robustness, manual checks were performed.

### Grain developmental time course

The *P1* NILs grown in 2018 and 2019 at Morley were used for the grain developmental time courses. For this, we tagged 70 ears per NIL over five replicated blocks in the field at ear emergence (spike fully emerged and peduncle just visible), as described in Brinton et al. (2017). Ten spikes per NIL, per block, were collected at five (2018) and six (2019) different timepoints. These were ear emergence, 3, 9, 16, and 22 dpa in 2018, while in 2019, the timepoints included ear emergence, anthesis (here measured as anther extrusion), 7, 14, 21, and 28 dpa. Grain measurements were performed as described in Brinton et al. (2017).

### Cell size measurements

We measured cell size of mature grains from *P1^WT^* and *P1^POL^* NILs collected from three field blocks grown at Morley in 2019. Within each block, we sampled three spikes and from each spike, we sampled grains from florets 2 and 4 of the two central spikelets. In total, this resulted in 18 grains per genotype per floret position (2 grains × 3 spikes × 3 field blocks). Dry grain samples were mounted crease-down onto 12.5 mm diameter aluminum pin stubs using double-sided 12 mm adhesive carbon discs (Agar Scientific Ltd, Stansted, Essex). The stubs were then sputter coated with ∼15-nm gold in a high-resolution sputter coater (Agar Scientific Ltd) and transferred to a Zeiss Supra 55 VP FEG scanning electron microscope (Zeiss SMT, Germany). The samples were viewed at 3 kV with a magnification of ×1,500 and digital TIFF files were stored. The surface of each grain was imaged in the top, middle and bottom thirds of the grain (excluding the embryo; Supplemental Figure S6) with three images taken in each section (nine images total per grain). Cell length was measured manually using the Fiji distribution of ImageJ (Schindelin et al., 2012). For statistical analyses, only images with ≥30 cell measurements were used. For each image, the median cell length was calculated. The image medians were then used to calculate a median cell length value for each section (bottom/middle/top) of each grain.

### Genetic mapping of *P1*

To fine-map *P1*, we first generated markers by performing exome-capture on *T. polonicum* accession T1100002, hexaploid wheat cultivar Paragon, and tetraploid wheat cultivar Langdon. These three samples were exome-sequenced in a pool of eight samples on a single Illumina HiSeq2000 lane following published protocols (Krasileva et al., 2017). This generated 27,919,048, 30,795,964, and 30,683,631 reads, respectively, which were mapped to the RefSeqv1.0 (International Wheat Genome Sequencing et al., 2018) assembly using bwa-0.7.15 (bwa mem -t 8 -M; Li and Durbin, 2009; Li, 2013). The resulting SAM file was converted to BAM format using samtools-1.3.1 (samtools view -b -h; Li et al., 2009) and sorted by chromosome position (samtools sort). Optical and PCR duplicates were marked using picard-1.134 (picard MarkDuplicates MAX_FILE_HANDLES_FOR_READ_ENDS_MAP = 1024 VALIDATION_STRINGENCY=LENIENT http://broadinstitute.github.io/picard/). SNPs were called for chromosome 7A with freebayes-1.1.0 (freebayes -0 -t; Garrison and Marth, 2012), and filtered using bcftools-1.3.1 (bcftools filter; Li and Durbin, 2009). Lastly, the vcf file was compressed with bgzip, indexed with tabix-0.2.6 (tabix -p vcf; Li and Durbin, 2009) before extracting relevant data in a user-friendly format with bcftools-1.3.1 (bcftools query -H -f ‘%CHROM\t%POS\t%REF\t%ALT{0}\t%QUAL\t%INFO/DP\t%INFO/RO\t%INFO/AO{0}[\t%GT\t%DP\t%RO\t%AO{0}]\n’). The SNPs were filtered for polymorphisms between *T. polonicum* and the two cultivars Paragon and Langdon. These putative SNPs were used to design KASP markers on chromosome 7A using PolyMarker (Ramirez-Gonzalez et al., 2015). KASP assays were validated in the parental NILs and then used for genetic mapping of *P1* as indicated in Supplemental Data Sets S7–S9.

We performed the mapping using a set of BC_4_ and BC_6_ recombinant inbred lines (RILs) derived from the *P1* NILs. In the first round we identified 17 BC_4_F_2_ heterozygous recombinant lines between markers *S1* and *S9.* We screened 12 BC_4_F_3_ progeny for each line to identify homozygous recombinants, which were phenotyped for glume length (Supplemental Data Set S7). To further define the *P1* interval, we screened an additional 1,867 BC_6_F_2_ plants heterozygous across the *S2* and *S7* interval. We identified 64 independent recombinants between markers *S2* and *S10*, which were phenotyped for glume length and genotyped with a further 21 markers (Supplemental Data Set S8). F_3_ progeny of the eight most critical recombinants (Supplemental Data Set S9) was grown at the John Innes Center Experimental trials site and phenotyped for height, grain weight, spike length, and grain morphometrics.

To test the isogenic nature of the BC_4_*P1* NILs, we used the Axiom 35k Breeders’ Array (Allen et al., 2017). The array showed that 98.7% of markers (32,839) were monomorphic between the NILs, with 418 polymorphisms between the NILs. More than 65% of the polymorphisms (272) were located on chromosome 7A, while the remaining were distributed evenly across other chromosomes.

We also mapped the *P1* locus in a cross between the *P1^POL^* NIL to hexaploid wheat variety “Chinese Spring”, which both share the same *VRT-A2* haplotype apart for the intron-1 sequence substitution (Supplemental Data Set S10). F_1_ plants were self-pollinated and we phenotyped and genotyped 84 F_2_ individuals from two independent F_1_ plants (168 F_2_ plants altogether) using eleven genetic markers on chromosome 7A (Supplemental Data Set S11). We constructed a genetic map using the genotype data and performed QTL analysis with the glume length phenotype using R/qtl (Broman et al., 2003); marker *SP1POL* had the highest LOD score (Supplemental Figure S9).

### PCR markers

The mapping populations were genotyped as described in Trick et al. (2012), with the following changes: 2 µL DNA (10–40 ng) was mixed with 2 µL of mastermix (2-µL PACE (Standard ROX; 3CR Bioscience) with 0.056-µL primer assay) for a total reaction volume of 4 µL. All KASP markers used for map-based cloning are listed in Supplemental Data Set S21. Standard PCR as well as RT-qPCR primers, their annealing temperatures and amplicon sizes are listed in Supplemental Data Set S22.

### *TraesCS7A02G175200* gene model

Using the expVIP browser (Borrill et al., 2016; Ramirez-Gonzalez et al., 2018), expression of *TraesCS7A02G175200* showed high expression in young seedlings (vegetative plants with 1-cm long spikes). The corresponding transcriptome data (Zadoks growth stage 30; Choulet et al., 2014) was downloaded, and aligned to the genomic RefSeqv1.0 assembly using HiSat2 v2.1.0 (hisat2 -p 16; Kim et al., 2015). The SAM file was converted to BAM format, sorted, and optical duplicates were marked as described for the exome capture data above. The depth of reads was measured using samtools-1.3.1 (samtools depth -a). The RNA-seq data supports the *TraesCS7A02G175200.1* gene model and its predicted untranslated regions Supplemental Figure S7B).

### Phylogenetic analysis of StMADS11-like family

Amino acid sequences of StMADS11 and related proteins were aligned in MEGA X using MUSCLE with default settings (Gap open penalty: -2.9; Gap extension penalty: 0; Hydrophobicity multiplier: 1.2; Clustering method: UPGMA; Edgar, 2004a, 2004b; Kumar et al., 2018; Schilling et al., 2020)). The alignment was then used to create a neighbor-joining tree in MEGA X (Test of phylogeny: 1,000 bootstraps; Model: Poisson; Rates among sites: Uniform; Gaps/Missing Data: Pairwise deletion; Zuckerkandl and Pauling, 1965; Felsenstein, 1985; Saitou and Nei, 1987; Kumar et al., 2018). A list of all proteins used for the alignment can be found in Supplemental Data Set S23. The alignment and tree files are deposited at Dryad (https://datadryad.org/stash/share/a6HM2SGbQyigaK7r2AnYc3TFao1kF9kN8C1QzScBsUU).

### Haplotype variation of *TraesCS7A02G175200*

We sequenced the promoter (2,299 bp), *TraesCS7A02G175200* genomic sequence (5,591 bp, exon and introns), and 1,857-bp downstream of the termination codon (9,747 bp) in the *P1^POL^* NIL using primers detailed in Supplemental Data Set S22. We also sequenced the 5,591-bp exon–intron sequences of *TraesCS7A02G175200* in six *T. polonicum*, two *T. petropavlovskyi*, and four “Arrancada” accessions (Supplemental Data Set S13) using primers listed in Supplemental Data Set S22.

We developed a PCR marker (primers S37_Fwd and S37_Rev; Supplemental Data Set S22) to determine the presence of either the 563-bp (*VRT-A2a* allele) or the 160-bp (*VRT-A2b* allele) intron-1 rearrangement in a large diversity panel. We used this marker to assay the intron-1 status of 70 wild emmer (*T. dicoccoides*), 103 hexaploid landraces, 4 durum, 23 *T. polonicum*, 2 *T. petropavlovskyi*, and 7 hexaploid “Arrancada” landrace accessions (Supplemental Data Sets S12 and S13). For ease of use, we developed a KASP marker (*SP1POL*; Supplemental Data Set S21) that can distinguish the *VRT-A2a* from the *VRT-A2b* allele. We validated the lines screened with the *S37* marker and screened a further 98 hexaploid UK cultivars, 60 European hexaploid cultivars, 17 tetraploid *T. durum* cultivars, 13 *T. polonicum* accessions, and 2 *T. petropavlovskyi* accessions. The total number of lines screened for the two alleles was 367 accessions: 70 *T. dicoccoides*, 21 *T. durum*, 268 *T. aestivum*, 36 *T. polonicum*, and 4 *T. petropavlovskyi*. We also used available genome sequences of 16 hexaploid (Walkowiak et al., 2020) and 3 tetraploid (Avni et al., 2017; Maccaferri et al., 2019; Walkowiak et al., 2020) cultivars and accessions to characterize *TraesCS7A02G175200* across its promoter, exon–intron sequences and 3′-untranslated region (Supplemental Data Set S10). We also evaluated the wider haplotype of the *P1* NILs, 16 hexaploid and 1 tetraploid cultivar, 7 *T. polonicum*, 2 *T. petropavlovskyi*, and 7 “Arrancada” landrace accessions using 14 markers spanning the 7A physical region (Supplemental Data Set S14). Details of the accessions used are listed in Supplemental Data Sets S12 and S13.

### Phylogenetic footprinting

We extracted intron-1 sequences of *VRT2* orthologs from barley (*HORVU7Hr1G036130*), *Brachypodium* (*Bradi1g45812*), rice (*Os06g0217300*), maize (*GRMZM5G814279*), and sorghum (*SORBI_3010G085400*) and used these, alongside *TraesCS7A02G175200*, as query sequences in the mVISTA program (http://genome.lbl.gov/vista/index.shtml).

### Intron-1 motif discovery

The phylogenetic footprinting analysis revealed two conserved sequence peaks between wheat, barley, *Brachypodium*, rice, maize, and sorghum. These sequences, plus some flanking sequence (71 and 84 bp for both regions, respectively), were aligned using T-Coffee with default settings (https://www.ebi.ac.uk/Tools/msa/tcoffee/; Notredame et al., 2000; Madeira et al., 2019). We defined the motifs using the following approach: a nucleotide was considered conserved if it was identical in five out of the six species (83%). A maximum of four nucleotides with lower conservation was tolerated, provided the neighboring sequences were again highly conserved (83%). This yielded 34- and 69-bp sequences, which were designated Motifs 1 and 2, respectively (Supplemental Data Set S15). When tolerating only a single nucleotide with low conservation (<83%), a highly conserved 16- and 20-bp sequence was detected within Motifs 1 and 2, respectively (Supplemental Data Set S15). In addition, within Motif 2, we detected a 6 bp highly conserved palindromic sequence that we designated “palindrome” (Supplemental Data Set S15). As there are no homologous sequences to the *VRT-A2b* allele 160-bp rearrangement, we divided the sequence into three “motifs”, constituting the two sections made of matching flanking DNA and the leftover sequence (Supplemental Data Set S16).

### Transcription factor binding site analysis of predicted motifs

To search for possible transcription factor binding sites within the predicted motif sequences, we used three different tools. The sequences were queried one by one using the Binding Site Prediction tool from PlantRegMap (http://plantregmap.gao-lab.org/binding_site_prediction.php) with default settings for the *A. thaliana*, *O. sativa*, and *Z. mays* databases, respectively (Tian et al., 2020). Next, we queried the *A. thaliana*, *O. sativa*, and *Z. mays* databases of PlantPan3.0 using the TF/TFBS Search tool with a q-value cut-off of 0.05. Lastly, we used the Tomtom tool of MEME Suite 5.2.0 (http://meme-suite.org/tools/tomtom) to query the *A. thaliana* DAP motifs database from O'Malley et al. (2016) as well as the “JASPAR CORE (2018) plants” database (Khan et al., 2018) with default settings.

### RNA extraction

BC_4_ NILs were grown in 11-cm^2^ pots (1-L volume) in “John Innes Cereal Mix” (40% Medium Grade Peat, 40% Sterilized Soil, 20% Horticultural Grit, 1.3 kg·m^−3^ PG Mix 14-16-18 + Te Base Fertiliser, 1 kg·m^−3^ Osmocote Mini 16-8-11 2 mg + Te 0.02% B, Wetting Agent, 3 kg·m^−3^ Maglime, 300 g·m^−3^ Exemptor) under long day conditions (16-h light: 8-h dark) in the glasshouse. Tissues from four to five biological samples per line and timepoint were harvested, immediately placed into 2-mL tubes in liquid nitrogen and stored at −80°C until needed. For meristem tissues, samples were dissected using a stereo microscope (Leica MZ16) and processed as above. Details of tissues sampled are presented in Supplemental Data Set S17. For T_0_ transgenic plants, we sampled flag leaves, glumes (central florets), and grains (florets 1 and 2) at 21 dpa. For T_1_ transgenic plants, we sampled flag leaves, glumes (central florets), grains (floret 2), and rachis at 14 dpa.

The grain and rachis samples were homogenized using mortar and pestle with liquid nitrogen. All other tissues were homogenized in a SPEX CertiPrep 2010-230 Geno/Grinder (Cat No.: 12605297, Fischer Scientific) using 5-mm steel beads (Cat No.: 69989, Qiagen); tubes were shaken in 20-s bursts at 1,500 r.p.m., then immediately transferred back into liquid nitrogen. Depending on the tissue type, this was repeated up to two times.

RNA was extracted using three different methods depending on the tissue:

- For young spikes (up until Floret primordium stage W3.5), we used the Qiagen RNeasy Plant Mini Kit (Cat No.: 74904, Qiagen) with RLT buffer according to the manufacturer’s protocol, as it enables recovery of RNA from small input samples. DNA digestion was performed using the RNase-free DNase set (Cat No.: 79254, Qiagen) according to the manufacturer’s protocol.
- For all other nongrain tissues, we used the Spectrum Plant Total RNA kit (Cat No.: STRN250-1KT, Sigma), following Protocol A of the manufacturer’s protocol and using 750 μL of binding solution. DNA digestion was performed using the on-column DNase I digestion set (Cat No.: DNASE70-1SET, Sigma) according to the manufacturer’s protocol.
- For grain samples, 500 μL of RNA extraction buffer (0.1-M Tris pH 8.0, 5-mM EDTA pH 8.0, 0.1-M NaCl, 0.5% SDS; autoclaved) with 1% β-Mercaptoethanol (Cat No.: M3148, Merck) and 100 μL of Ambion Plant RNA Isolation Aid (Cat No.: AM9690, Thermofisher) were added to each sample, before vortexing. Tissue debris as well as polysaccharides and polyphenols were pelleted at 13,000 r.p.m. for 10 min in a microcentrifuge. The supernatant was transferred to a new 1.5-mL tube, before adding 500 μL of acid-phenol:chloroform:IAA (125:24:1; Cat No.: AM9720, Thermofisher). The tubes were shaken in a SPEX CertiPrep 2010-230 Geno/Grinder for 10 min at 500 r.p.m., and then placed in a microcentrifuge at 13,000 r.p.m. for 15 min to separate the organic and aqueous components. The supernatant (aqueous phase) was transferred to a new 1.5-mL tube with 500 μL of chloroform (Cat No.: C/4960/PB17, FisherScientific). The tubes were inverted 10 times and then placed in a microcentrifuge for 15 min at 13,000 r.p.m. The supernatant was transferred to a new 1.5 mL tube with 360 μL of Isopropanol (Cat No.: P/7500/PC17, FisherScientific) and 45-μL 3-M sodium acetate (pH 5.2). The tube was inverted 10 times to mix the solution, before placing at 4°C for 1 h to precipitate RNA. The RNA was pelleted in a microcentrifuge at 4°C by spinning for 30 min at 13,000 r.p.m. The supernatant was carefully tipped off to not lose the pellet. The tubes were then washed twice with 70% ethanol (Cat No.: 20821.330, VWR) and centrifuged between washes at 13,000 r.p.m. for 5 min at 4°C. The supernatant was then carefully discarded and remaining droplets of Ethanol removed using a pipette tip, before adding 100 μL of nuclease-free water (Cat No.: AM9937, Thermofisher).

### RT-qPCR

RNA was reverse transcribed using M-MLV reverse transcriptase (Cat No.: 28025013, Thermofisher) according to the manufacturer’s protocol. For the RT-qPCR reactions, LightCycler 480 SYBR Green I Master Mix (Roche Applied Science, UK) was used according to the manufacturer’s protocol. The reactions were run in a LightCycler 480 instrument (Roche Applied Science, UK) under the following conditions: 5 min at 95°C; 45 cycles of 10 s at 95°C, 15 s at 62°C, 30 s at 72°C; dissociation curve from 60°C to 95°C to determine primer specificity. All reactions were performed with three technical replicates per sample and using *TaActin* as reference gene (Li et al., 2019). Relative gene expression was calculated using the 2^−ΔΔCt^ method (Livak and Schmittgen, 2001) with a common calibrator so that values are comparable across genes, tissues, and developmental stages. All primers used in RT-qPCR are listed in Supplemental Data Set S22.

### Construct assembly

A modified version of the GoldenGate (MoClo) compatible level 2 vector pGoldenGreenGate-M (pGGG-M) as described in Hayta et al. (2019) was used in this study. The pGGG-AH-L2P2 acceptor plasmid is comprised of the hygromycin resistance gene (hpt) containing the Cat1 intron driven by the rice actin1 (*OsAct1*) promoter for in planta selection and a LacZ-MCS flanked by two *Bsa*I sites at MoClo position 2 with standardized overhangs to accept basic (level 0) components. In brief, the *T. polonicum VRT-A2* promoter (2,299 bp), genomic sequence (5,585 bp), 1,000-bp downstream of STOP codon, and NOS terminator (8,916 bp total) were cloned into pGGG-AH-L2P2 using standard Golden Gate MoClo assembly (Werner et al., 2012), resulting in construct pGGG-AH-VRT-A2 (Supplemental Figure S11). Several *Bsa*I and *Bbs*I sites had to be domesticated to make the *T. polonicum VRT-A2* sequence suitable for Golden Gate MoClo assembly, including three sites in the promoter (C4174T, G4549A, C5755T), one site in exon-1 (C6126T; V47V), one site in an intronic MITE (C8377T), and one site in exon-3 (T9477C; L106L). Six nucleotides from a partial LINE in intron-5 were omitted by mistake from the genomic sequence (hence 5,585 bp from start to termination codon instead of 5,591 bp). The construct was electroporated into the hypervirulent *Agrobacterium tumefaciens* strain AGL1 (Lazo et al., 1991) containing the helper plasmid pAL155 (additional *Vir*G gene). Standard inoculums of *Agrobacterium* (Tingay et al., 1997) were prepared as described in Hayta et al. (2019).

### Wheat transformation

Hexaploid wheat c.v. “Fielder” was transformed using the previously described method by Hayta et al. (2019). In brief, under aseptic conditions wheat immature embryos were isolated, pretreated by centrifugation, inoculated with *A*. *tumefaciens* AGL1 containing pGGG-AH-VRT-A2 and co-cultivated for 3 days. Wheat callus proliferation, shoot regeneration, and rooting were carried out under a stringent hygromycin selection regime before the regenerated plantlets were transferred from in vitro to soil and acclimatized. Transgenesis was confirmed by *hpt* gene PCR; transgene copy number analysis was performed using Taqman qPCR and probe (Hayta et al., 2019). The values obtained were used to calculate copy number according to published methods (Livak and Schmittgen, 2001). Based on this copy number determination, we defined T_0_ lines as zero, low (one to two copies of pGGG-AH-VRT-A2), medium (four to five copies of pGGG-AH-VRT-A2), and high (nine or more copies of pGGG-AH-VRT-A2) copy number lines (Supplemental Data Set S18). For T_1_ plants, we determined the presence/absence of the transgene using the *SP1POL* marker and maintained each plants’ copy-number classifications based on their mother T_0_ plant.

### Statistical analyses

#### Field experiments

To determine the differences between the *P1^POL^* and *P1^WT^* NILs, we performed analysis of variance (ANOVA) on the multiple field phenotypic data in RStudio (v1.3.1056). For the overall analysis we included block (nested in location), genotype, location, and the genotype*location interaction in the model. For the analysis of individual locations, we used a simple two-way ANOVA including block and genotype. For the BC_6_ RILs, we determined the effect of the *VRT-A2* allele on height, TGW, spike length, grain width, and length using a two-way ANOVA using block and the *VRT-A2* genotype in the model. For glume length, RILs were assigned as having a normal or long-glume phenotype using a post hoc Dunnett’s test to compare with the *P1^POL^* and *P1^WT^* controls.

#### Spike dissection

We used glume measurements from spikelets 1 (basal) to 20 (apical) to determine the differences between the *P1^POL^* and *P1^WT^* NILs. We did not include datapoints from spikelets 21 to 23 as these were only found in very few instances (balanced between genotype) and made up <2.5% of the total grain data (50 datapoints excluded from 2,117 total grain values). Likewise, we did not include 80 datapoints from florets 5, which between them represented 3.8% of grain data and were found only in spikelets 4–13 in both NILs. Given that the experimental unit is the field plot to which the genotype was randomized within each block, we analyzed the data using a split-plot ANOVA in which the “Spikelet” was nested within the “Genotype*Block” interaction. The ANOVA therefore included the following terms: Block, Genotype, Spikelet, and Genotype*Spikelet interactions, with *F*-statistic and *P-*values calculated based on the “Block*Genotype” error term (for Block and Genotype) or the Residual error term for the other factors.

We analyzed the grain, lemma, and palea data from spikelets 1 to 20 and florets 1–4 to determine the differences between the *P1* NILs. Given that the florets are nested in the spikelet, and the spikelet is nested within the genotype*block interaction (i.e. the florets and spikelets are not randomly assigned), we analyzed the data as a split–split plot design using the corresponding error terms for calculating the *F*-statistic and *P-*values. This model included the block, genotype, spikelet, floret, and corresponding interaction terms. We also performed individual ANOVAs for each floret position using the same model as above, with the exception that we excluded floret as a factor.

#### Grain development time course

Each block at every timepoint consisted of approximately 100 grains (10 spikes × 10 grains) per NIL. The grain morphometrics were averaged across the 100 grains (as they were considered to be subsamples) to yield a single value per timepoint, resulting in five datapoints per NIL per timepoint. We performed a two-way ANOVA with Block and Genotype in the model to determine whether *P1* affects grain morphometrics at the sampled timepoints.

#### Cell size measurements

We analyzed the data independently for florets 2 and 4 using a three-way ANOVA including Block, Genotype, Block*Genotype, Section, and the Genotype*Section interaction. Given the significant Genotype*Section interactions, we explored differences between *P1* genotypes for each section using Tukey multiple comparison as implemented in RStudio (v1.3.1056).

*Expression*: We evaluated differences in expression levels of *VRT2* and *MADS22* homoeologs by performing *t* tests between the 2^−ΔΔCt^ expression values of *P1^POL^* and *P1^WT^* NILs for each individual tissue*timepoint comparison.

#### Phenotypes in T_0_ and T_1_ transgenic lines

To evaluate differences in phenotype between the four categories of transgenic lines (zero, low, medium, and high copy number lines; Supplemental Data Set S19), we performed one-way ANOVAs for each floret position including “transgene copy number” as the single factor. This was done independently for each generation of transgenic lines. Given that “transgene copy number” was significant for all phenotypes (glume, lemma, palea, and grain length) and across all florets, we performed Tukey multiple comparison tests to determine differences between the four “transgene copy number” categories as well as Dunnett tests against the zero copy number control lines (Supplemental Data Set S19).

#### Correlation of phenotype and expression in transgenic T_0_ and T_1_ lines

For each generation of transgenic lines, we calculated the Pearson’s correlation coefficient between *VRT-A2* expression (in flag leaf, glume, grain, and rachis [only T_1_]) and phenotypic traits (internode length (only T_0_), spike length, glume length, lemma length, grain length, palea length) in R (Supplemental Data Set S20). We used geom_smooth(method = “lm”) to plot the regression line and 95% confidence interval.

### Accession numbers

Sequence data from this article can be found in the EMBL/GenBank data libraries under accession numbers MW289820–MW289831 (*TraesCS7A02G175200* sequence for *T. polonicum*, *T. petropavlovskyi*, and Arrancada accessions), and MW307955–MW307976 for *T. dicoccoides* and Watkins accessions (Supplemental Data Sets S12 and S13). Construct pGGG-AH-VRT-A2 has been deposited in Addgene (ID 163703) and the sequence is available (NCBI accession MW289819). The raw exome capture reads used in this study are available from NCBI BioProject PRJNA684023. Seed of the BC_6_*P1^POL^* NIL is available from the JIC GRU (Code WM0013). Full-size images of spikes and spikelets of the *T. polonicum*, *T. petropavlovskyi*, and “Arrancada” accessions used in this study and in Figure 3, B and C are deposited at Dryad (https://datadryad.org/stash/share/a6HM2SGbQyigaK7r2AnYc3TFao1kF9kN8C1QzScBsUU).

## Supplemental data

The following materials are available in the online version of this article.

**Supplemental Figure S1.** (Supports Figures 1 and 2). Genotyping of BC_4_*P1* NILs using Breeders’ 35K Axiom Array.

**Supplemental Figure S2.** (Supports Table 1). Length of internodes, peduncles, and spikes in *P1^WT^* (*N* = 7) and *P1^POL^* (*N* = 5) NILs grown in the field in 2017.

**Supplemental Figure S3.** (Supports Figure 1). Time course tracking ovary/grain length and width of field-grown *P1^WT^* and *P1^POL^* NILs from 2018 (A, B) and 2019 (C).

**Supplemental Figure S4.** (Supports Figure 1). Glume width along spikes of *P1^WT^* and *P1^POL^* NILs. Positions are numbered from basal to apical spikelets.

**Supplemental Figure S5.** (Supports Figure 1). Lemma width (A), grain width (B), palea length (C), and palea width (D) at each floret position along *P1^WT^* and *P1^POL^* NILs spikes.

**Supplemental Figure S6.** (Supports Figure 1). Pericarp cell length in *P1* NILs. (A) Illustration of grain sections used for SEM, including bottom (B), middle (M), and top (T) sections.

**Supplemental Figure S7.** (Supports Figures 2 and 3). Characterization of *P1* critical interval.

**Supplemental Figure S8.** (Supports Figure 4). Phylogenetic tree of StMADS11-like proteins from dicots and monocots made using neighbor-joining method and rooted at the midpoint (Saitou and Nei, 1987).

**Supplemental Figure S9.** (Supports Figures 2 and 3). QTL mapping and graphical genotype for the F_2_ population between Chinese Spring and the *P1^POL^* NIL.

**Supplemental Figure S10.** (Supports Figures 2 and 3). Examination of the 160-bp rearrangement within the *VRT-A2b* allele.

**Supplemental Figure S11.** (Supports Figure 5). *VRT-A2* complementation construct.

**Supplemental Figure S12.** (Supports Figure 5). Analysis of T_0_ transgenic lines for spike, glume, lemma, and grain length and correlation of spike and glume length with *VRT-A2* expression in glumes.

**Supplemental Figure S13.** (Supports Figure 5). Pearson correlations between *VRT-A2* relative expression in the glume at 14 dpa and glume length including the single outlier value which was not depicted in Figure 5D (*n* = 40 plants).

**Supplemental Data Set S1.** Field evaluation of *P1* NILs across multiple years and environments.

**Supplemental Data Set S2.** Effect of *P1* on glume area, length and width in *P1^POL^* and *P1^WT^* NILs.

**Supplemental Data Set S3.** Effect of *P1* on area, length and width (columns) of *P1^POL^* and *P1^WT^* NILs grain, lemma and palea (rows).

**Supplemental Data Set S4.** Grain morphometric values from CT-scans of 15 spikes of *P1^POL^* and 15 spikes of *P1^WT^* NILs collected at harvest ready (HR) stage.

**Supplemental Data Set S5.** Effect of *P1* on ovary/grain area, width and length (columns) in *P1^POL^* and *P1^WT^* NIL s grown in the field in 2018 and 2019.

**Supplemental Data Set S6.** Pericarp cell length of *P1^POL^* and *P1^WT^* NIL grains from florets 2 and 4.

**Supplemental Data Set S7.** Graphical genotype of BC_4_F_3_ homozygous RILs.

**Supplemental Data Set S8.** Graphical genotype of BC_6_F_2_ heterozygous RILs from second screen.

**Supplemental Data Set S9.** Graphical genotype and phenotype of BC_6_ RILs.

**Supplemental Data Set S10.** Haplotype analysis of *VRT-A2* (*TraesCS7A02G175200*) in *T. polonicum*, and genome sequenced tetraploid (Kronos, Svevo, Zavitan) and hexaploid (all others) wheat accessions/cultivars.

**Supplemental Data Set S11.** Graphical genotype and phenotype of F_2_ population from the Chinese Spring x *P1^POL^* NIL cross.

**Supplemental Data Set S12.** Wild emmer (*T. dicoccoides*), Durum (*T. durum*) and hexaploid Watkins landraces, UK recommended list cultivars, and Gediflux cultivars used in this study.

**Supplemental Data Set S13.** *Triticum polonicum*, *T. aestivum* (“Arrancada”), and *T. petropavlovskyi* accessions used in this study.

**Supplemental Data Set S14.** Haplotype of germplasm used in this study, as well as from the fully sequenced pangenome collection, across chromosome 7A including the *P1* locus.

**Supplemental Data Set S15.** Analysis of *VRT-A2* intron-1 conserved sequences across grass species.

**Supplemental Data Set S16.** Analysis of putative transcription factor binding sites within *VRT-A2* intron-1.

**Supplemental Data Set S17.** Expression data of *VRT2* and *TaSVP1* homoeologs in *P1^POL^* and *P1^WT^* NILs across different tissues and developmental stages.

**Supplemental Data Set S18.** Expression data of *VRT2* homoeologs in *P1* transgenic T_0_ lines in flag leaves, glumes and grains, and *VRT-A2* expression in T_1_ lines in flag leaves, glumes, grains, and rachis.

**Supplemental Data Set S19.** Effect of *P1* transgene copy number on spike, glume, lemma, palea, and grain length in T_0_ (*n* = 14) and T_1_ (*n* = 40) transgenic lines.

**Supplemental Data Set S20.** Correlation analysis between *VRT-A2* expression in transgenic T_0_ and T_1_ lines (Supplemental Data Set S18) with respect to phenotypic values (Supplemental Data Set S19).

**Supplemental Data Set S21.** List of KASP markers used to map the *P1* locus in mapping populations.

**Supplemental Data Set S22.** List of standard PCR primers as well as RT-qPCR primers used in this study.

**Supplemental Data Set S23.** List of StMADS11 family members used to create phylogenetic tree of *AtSVP* orthologs in grasses.

## Supplementary Material

koab119_Supplementary_Data

## Notes

J.S., N.M.A., and C.U. conceived the study. J.S. generated the NILs and mapping populations together with PS. N.M.A. performed most experiments. A.P. and O.H. genotyped Watkins, *T. dicoccoides*, and *T. polonicum* accessions using a PCR marker and sequenced amplicons. Y.C. helped with the analysis of *VRT-A2* alleles and haplotypes. A.E.B. dissected spikelets of NILs, phenotyped and assessed the data. The grains of these lines were imaged by J.F.B. and E.B. using scanning electron microscopy, with J.F.B. analyzing the data. M.S. designed and created the pGGG-AH-VRT-A2 construct; S.H. transformed cv Fielder with the construct, cultivated the plants and performed copy number analysis on them. J.S. performed field experiments and phenotyping. T.F. and P.C. helped with plant husbandry, phenotyping, and data collection. M.C., J.D., and C.N. performed CT-scans of field-grown spikelets and analyzed the data. J.F.B., N.M.A., and C.U. created the figures. C.U. performed statistical analysis of all data. N.M.A. and C.U. wrote the manuscript. All authors have read and approved the manuscript.

The author responsible for distribution of materials integral to the findings presented in this article in accordance with the policy described in the Instructions for Authors (https://academic.oup.com/plcell) is: Cristobal Uauy (cristobal.uauy@jic.ac.uk).
