# Supplementary material for: Ectopic expression of Triticum polonicum VRT-A2 underlies elongated glumes and grains in hexaploid wheat in a dosage-dependent manner
Source: Plant Cell. 2021 May 1;33(7):2296–319. doi: 10.1093/plcell/koab119 (PMC8364232; doi:10.1093/plcell/koab119)
Supplement: koab119_Supplementary_Data [file koab119_supplementary_data.zip › tpc.01033.2020-s01.pdf]

Supplemental Figures

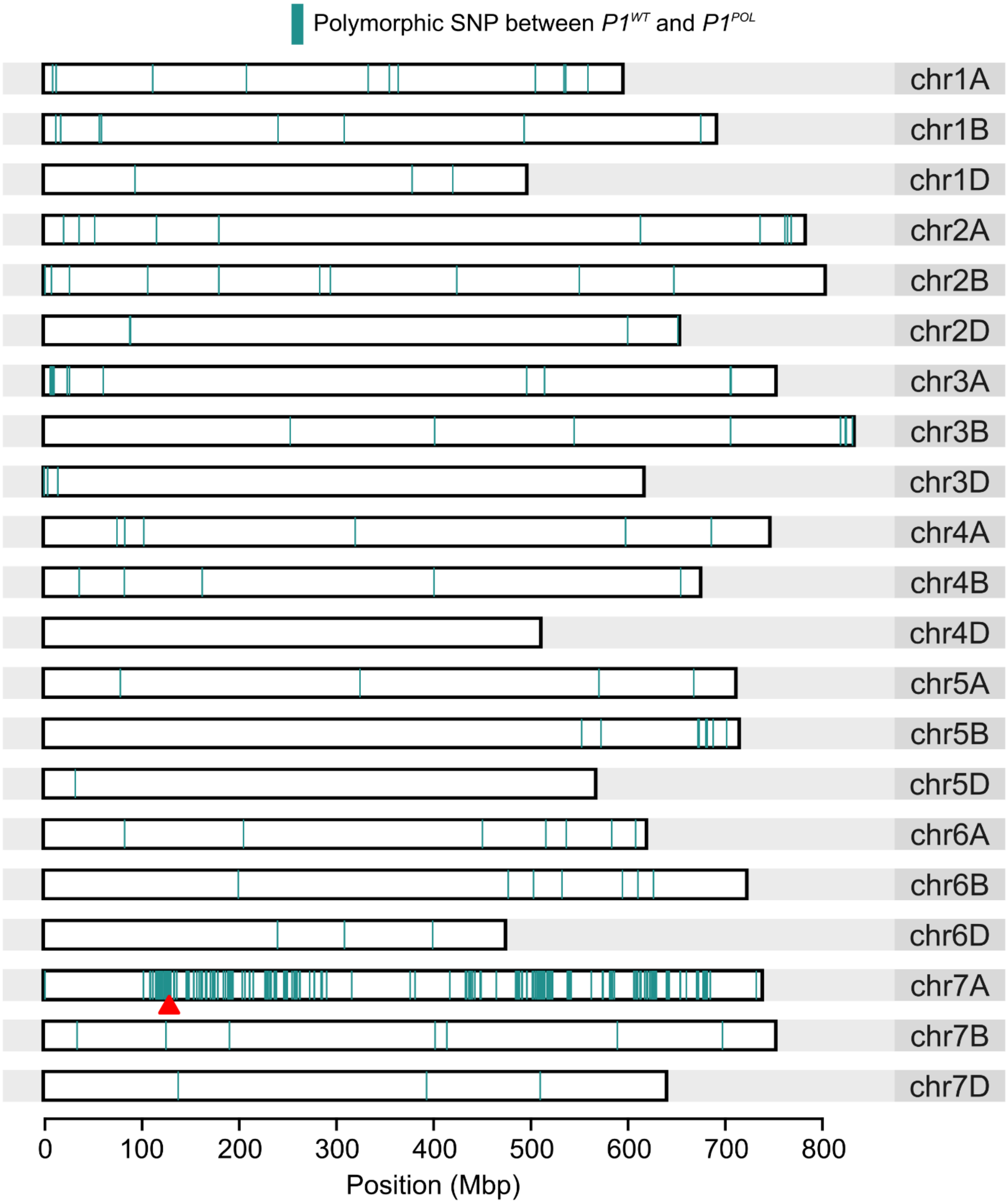

Supplemental Figure S1 (Supports Figure 1, 2): Genotyping of BC_4 *P1* NILs using Breeders' 35K Axiom Array. Polymorphic markers between *P1*^{WT} and *P1*^{POL} are indicated as green lines. Physical positions of markers were determined using the IWGSC RefSeqv1.0 assembly. The position of *VRT-A2* is marked by a red triangle.

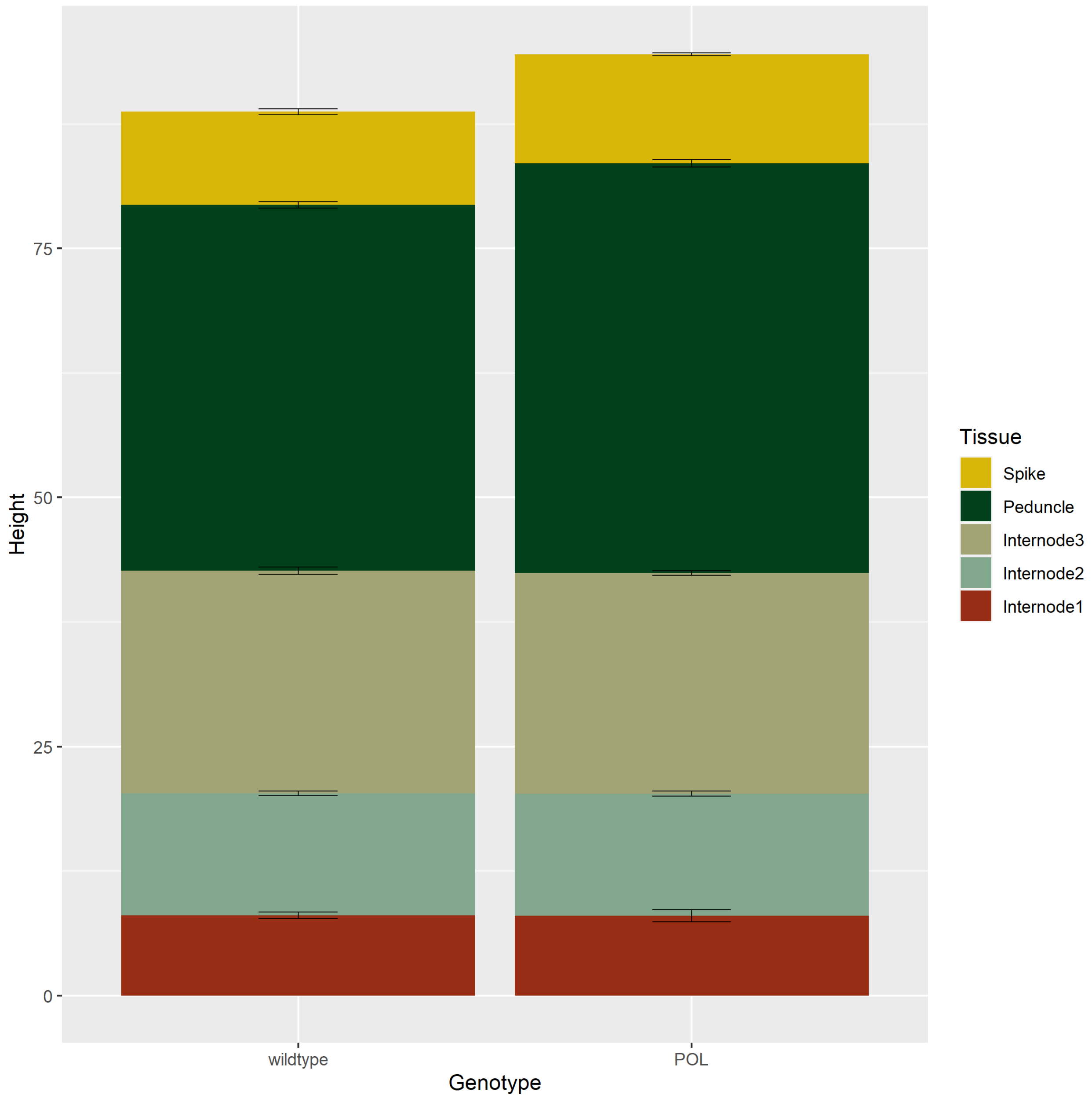

Supplemental Figure S2 (Supports Table 1)**:** Length of internodes, peduncles, and spikes in *P1*^{WT} (N=7) and *P1*^{POL} (N=5) NILs grown in the field in 2017. The three internodes are not significantly different between the NILs, whereas the peduncle is significantly different ($P < 0.001$) and the spike, in this experiment, is borderline ($P < 0.06$) non-significant. Error bars represent mean ± SEM.

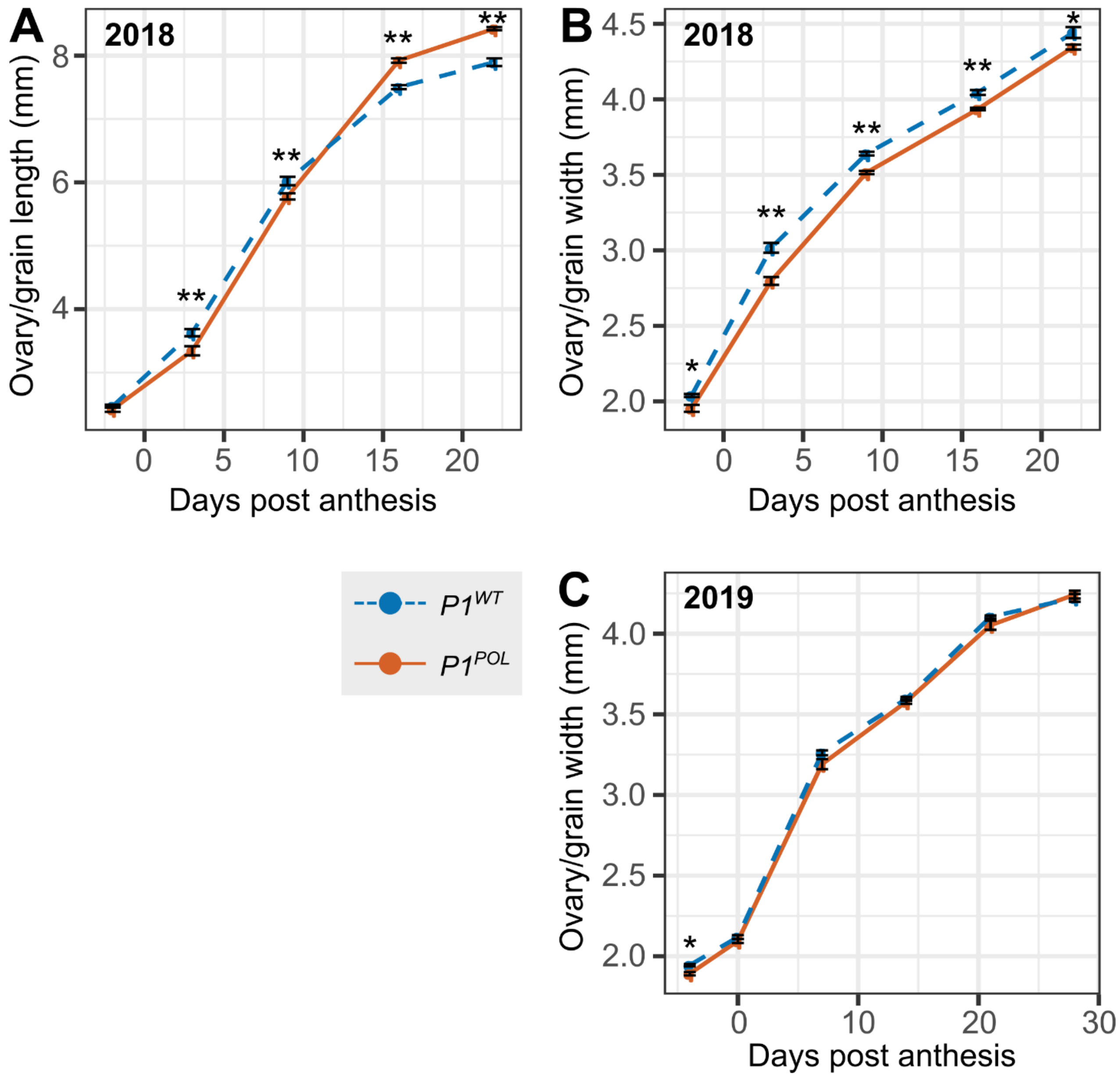

Supplemental Figure S3 (Supports Figure 1): Timecourse tracking ovary/grain length and width of field-grown *P1*^{WT} and *P1*^{POL} NILs from 2018 (**A, B**) and 2019 (**C**). Length from 2019 is presented in Figure 1D (N=50 per timepoint per genotype). Error bars represent mean ± SEM. *, *P* < 0.05; **, *P* < 0.01; ***, *P* < 0.001.

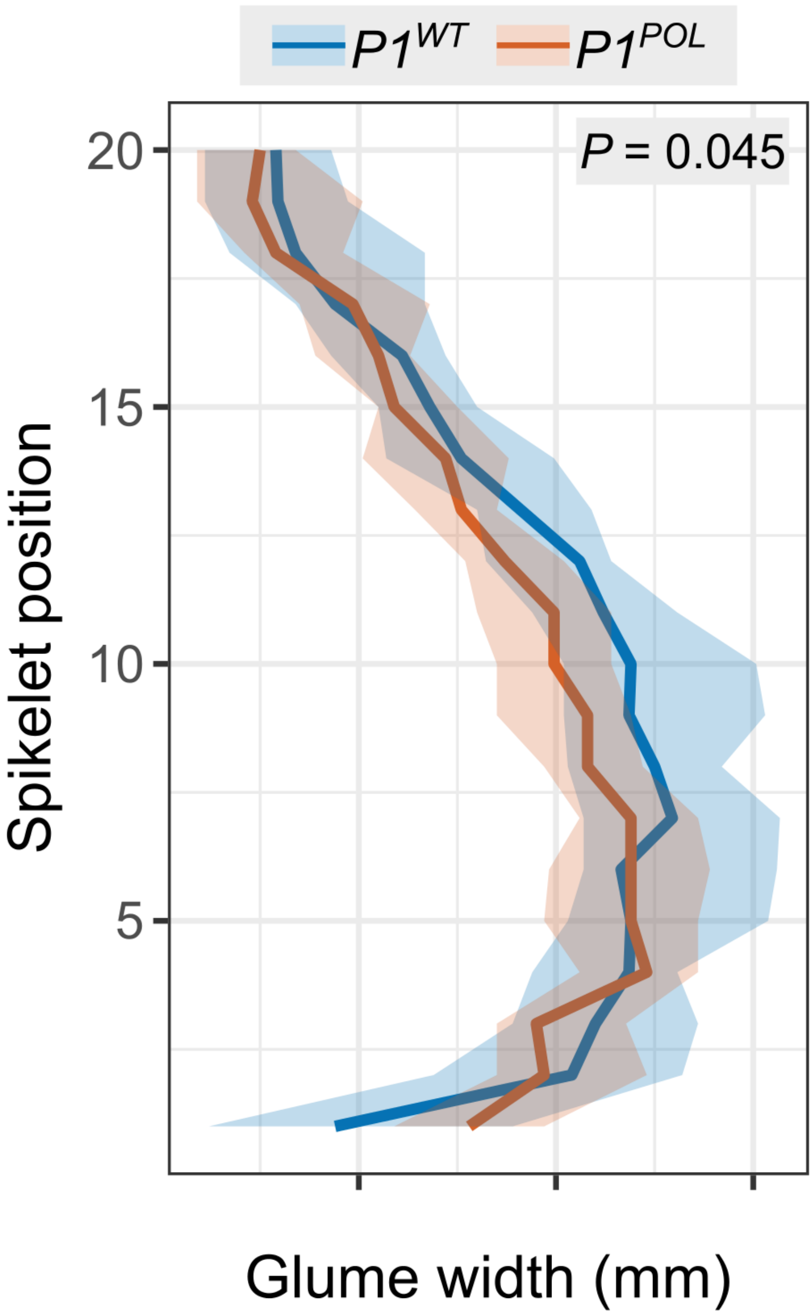

Supplemental Figure S4 (Supports Figure 1): Glume width along spikes of *P1*^{WT} and *P1*^{POL} NILs. Positions are numbered from basal to apical spikelets. Bold line represents the median value, ribbon represents the interquartile range (N=15 spikes).

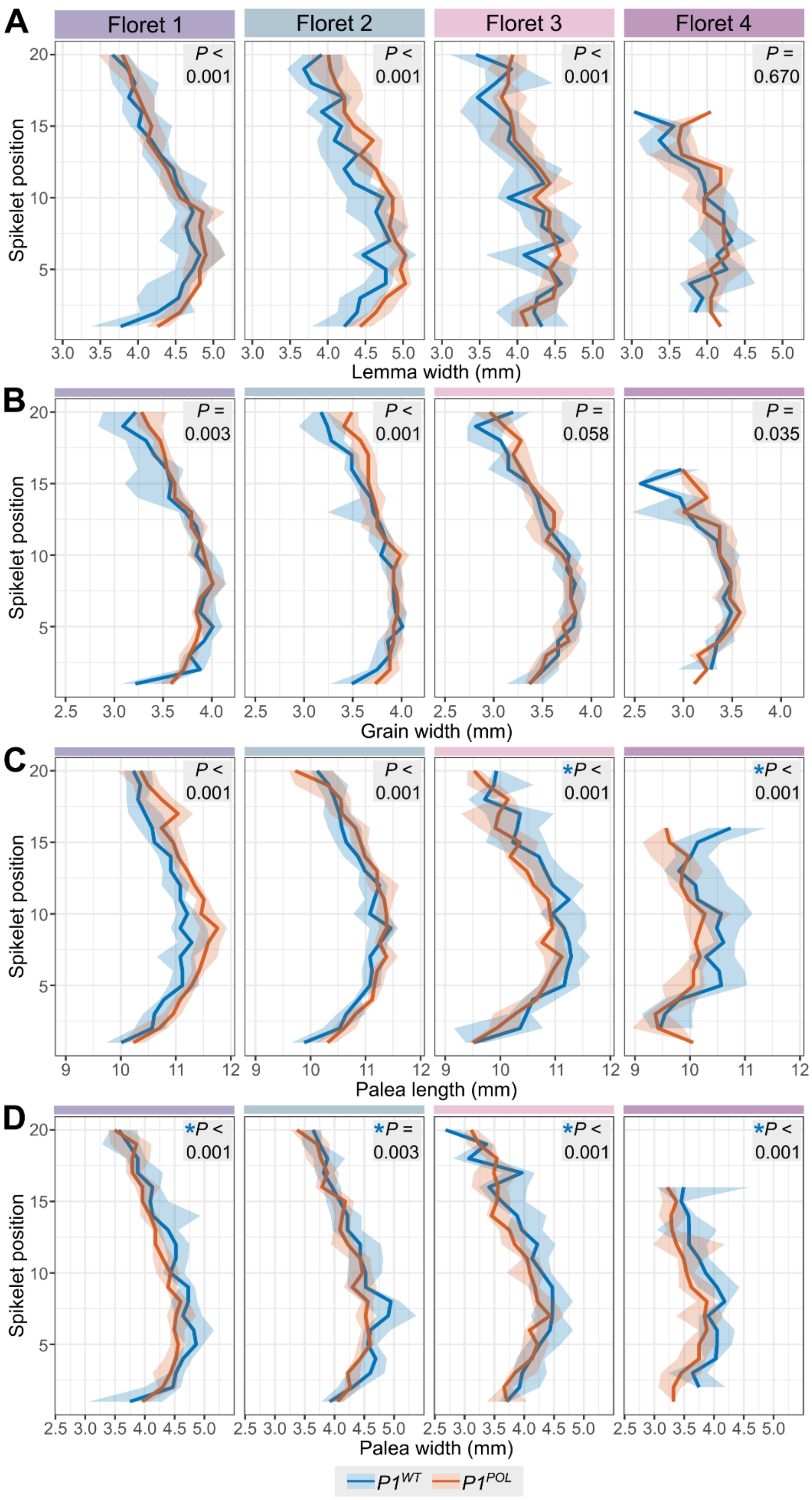

Supplemental Figure S5 (Supports Figure 1)**:** Lemma width (**A**), grain width (**B**), palea length (**C**), and palea width (**D**) at each floret position along *P1*^{WT} and *P1*^{POL} NILs spikes. Spikelet positions are numbered from basal to apical spikelets. Bold line represents the median value, ribbon represents the interquartile range (N=15 spikes).

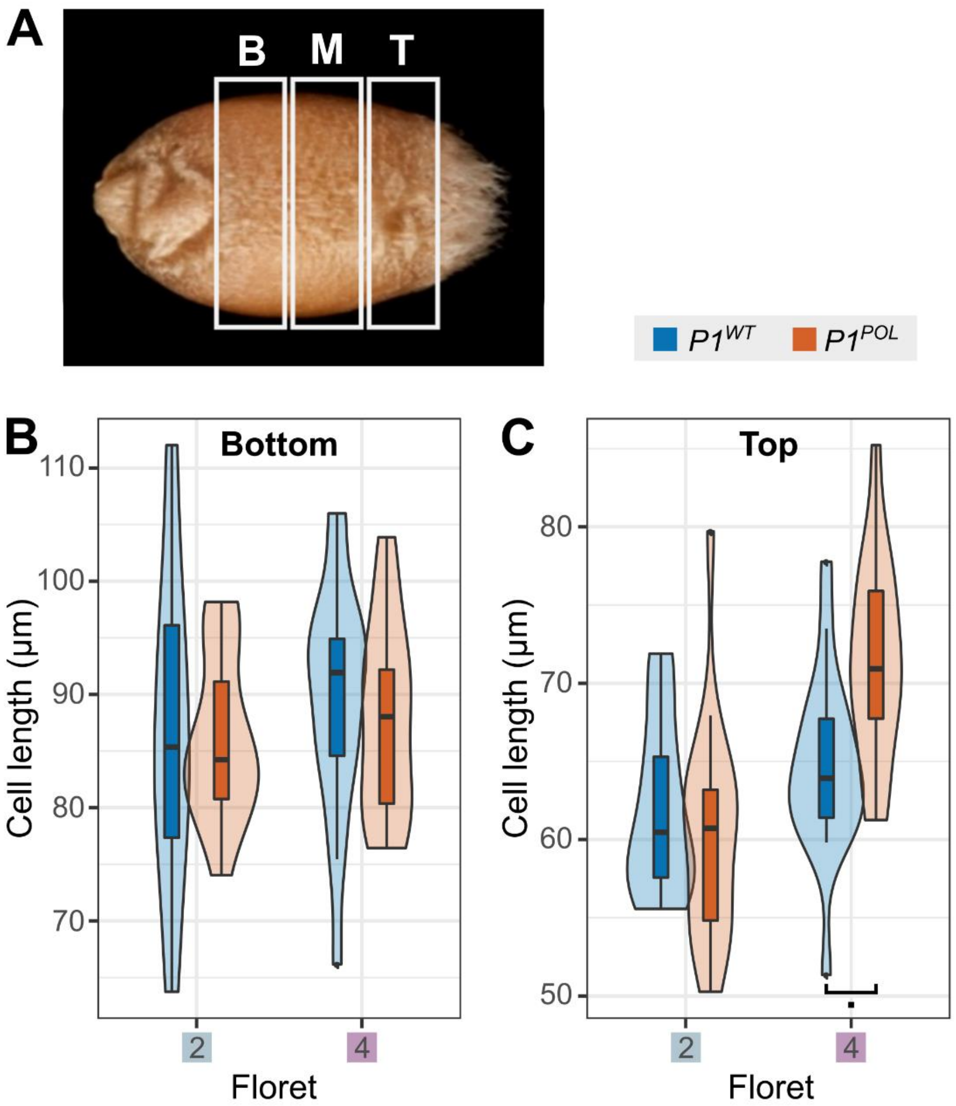

Supplemental Figure S6 (Supports Figure 1): Pericarp cell length in *P1* NILs. (**A**) Illustration of grain sections used for SEM, including bottom (B), middle (M) and top (T) sections. Pericarp cell length from bottom (**B**) and top (**C**) sections of grains from floret 2 and floret 4 for the $P1^{WT}$ and $P1^{POL}$ NILs (n= 18 grains). In (B) and (C), the box represents the middle 50% of data with the borders of the box representing the 25th and 75th percentile. The horizonal line in the middle of the box represents the median. Whiskers represent the minimum and maximum values, unless a point exceeds 1.5 times the inter-quartile range in which case the whisker represents this value and values beyond this are plotted as single points (outliers). *, $P < 0.05$.

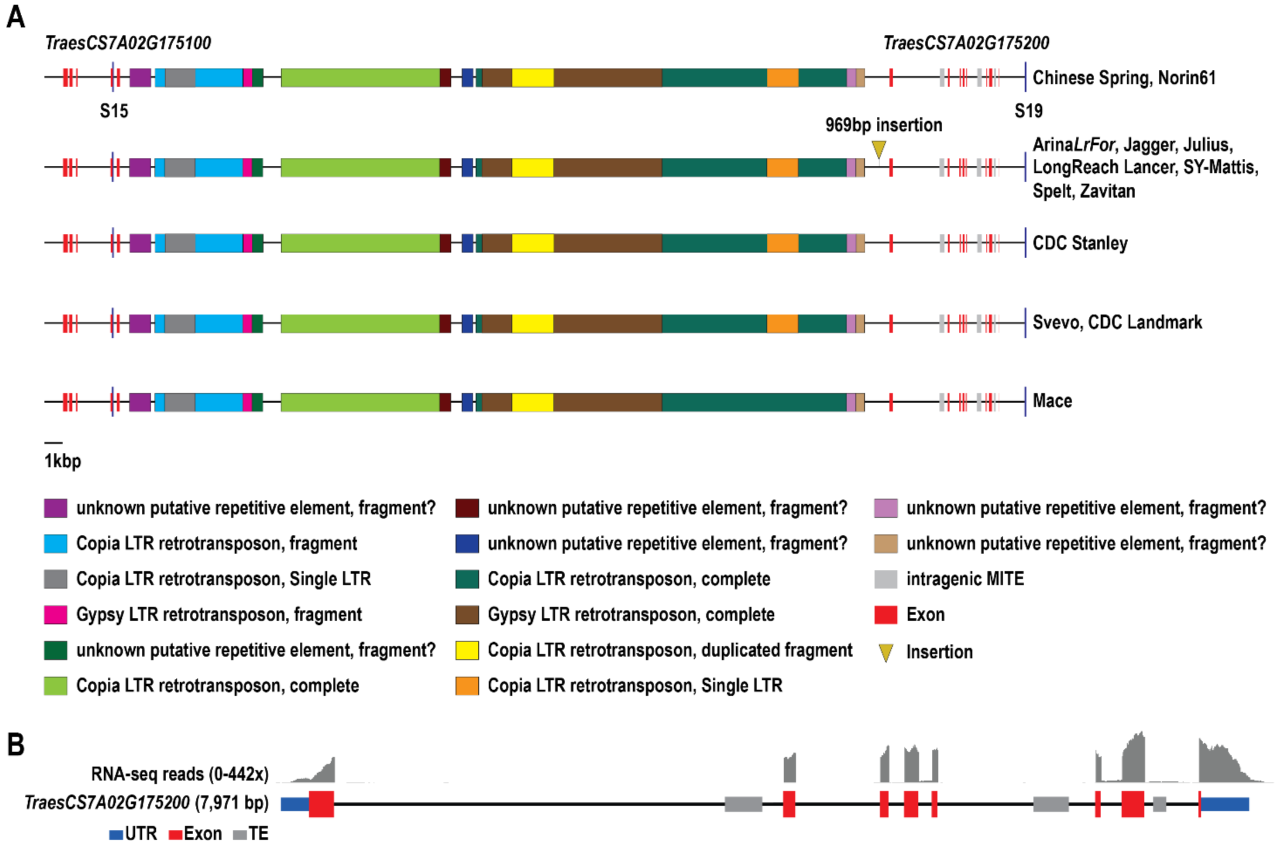

Supplemental Figure S7 (Supports Figure 2, 3): Characterisation of *P1* critical interval. (**A**) Annotation of physical interval between markers *S15* and *S19* in pangenome cultivars (Walkowiak *et al.*, 2020). (**B**) RNA-Seq coverage of *TraesCS7A02G175200* across predicted exon-intron structure.

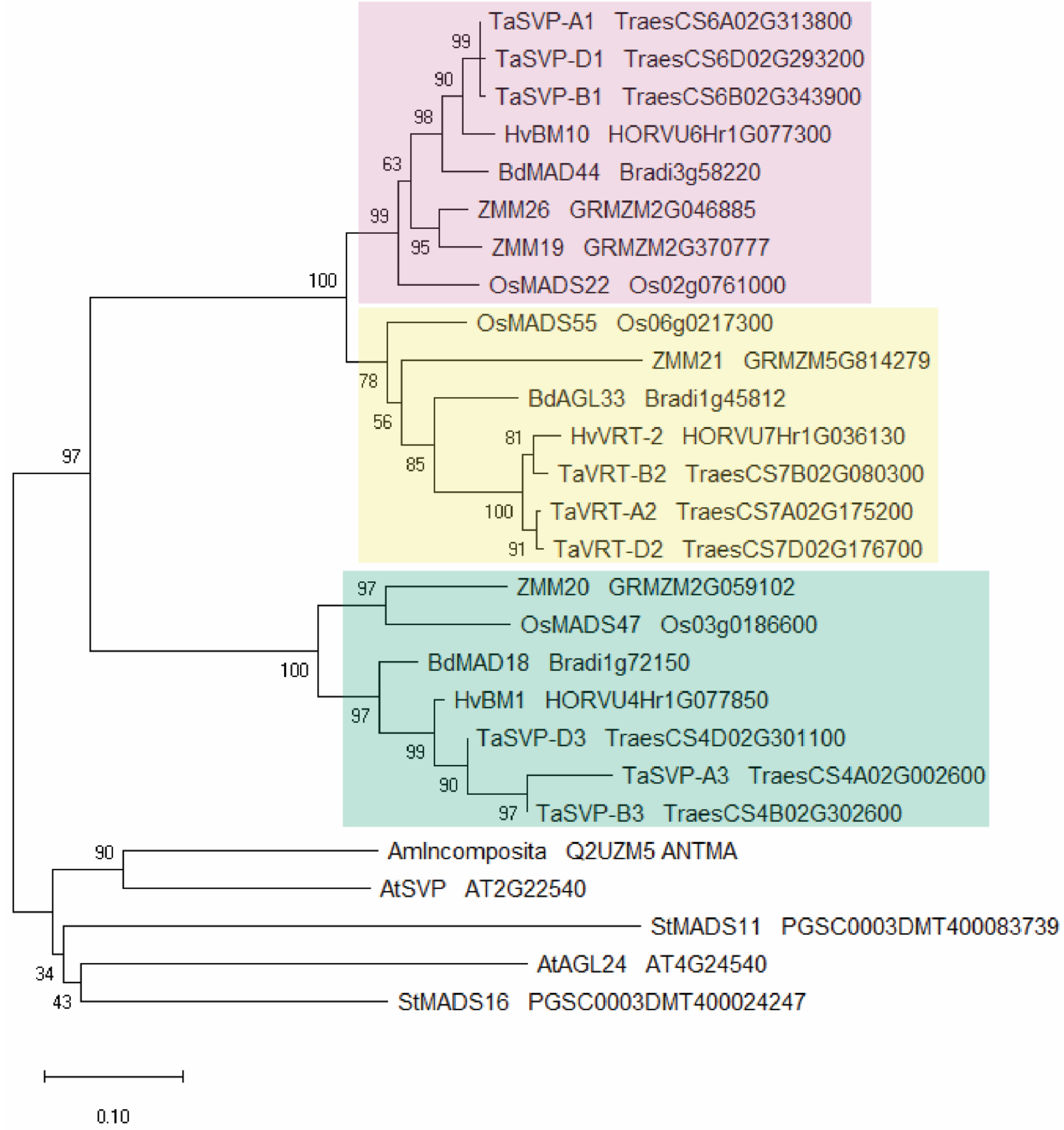

Supplemental Figure S8 (Supports Figure 4): Phylogenetic tree of StMADS11-like proteins from dicots and monocots made using neighbor-joining method and rooted at the midpoint (Saitou and Nei, 1987). Three SVP-like genes are present in grasses (four in maize), with VRT2 (yellow box) and TaSVP1 (pink box) being more similar to each other than to TaSVP3 (cyan box; (Schilling *et al.*, 2020)). Numbers next to branches indicate the percentage of replicate trees in which the associated proteins clustered together in the bootstrap test (1000 replicates; (Felsenstein, 1985)). Branch lengths were calculated using the Poisson correction method in units of amino acid substitutions per site (Zuckerkandl and Pauling, 1965).

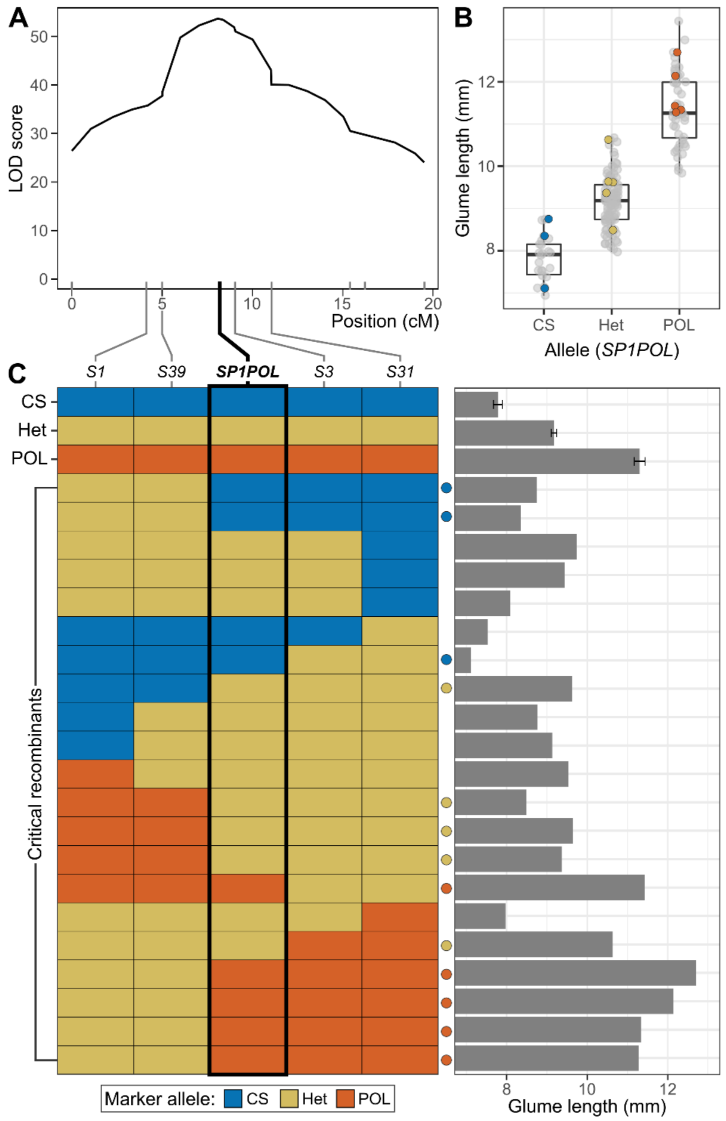

Supplemental Figure S9 (Supports Figure 2, 3): QTL mapping and graphical genotype for the F_2 population between Chinese Spring and the *P1*^{POL} NIL. (**A**) QTL plot of glume length for the 168 F_2 plants based on 11 genetic markers on chromosome 7A. (**B**) Box plots of glume length distribution of F_2 plants based on their genotype at the *SP1POL* marker (peak marker in the QTL plot in **A**); Chinese Spring (CS, blue), heterozygous (Het, gold), *P1*^{POL} (POL, orange/red). Coloured circles represent F_2 plants with a recombination event on either side of the *SP1POL* marker. This highlights their correct assignment to phenotypic classes based on the *SP1POL* marker genotype. The box represents the middle 50% of data with the borders of the box representing the 25th and 75th percentile. The horizonal line in the middle of the box represents the median. Whiskers represent the minimum and maximum values. (**C**) Graphical genotype and phenotype from the CS x POL F_2 population between markers *S1* and *S31*. F_2 plants which were fixed across this interval were classified based on their genotype and their average glume length is shown in the top three rows (CS= 18 F_2 plants, Het = 86 F_2 plants, POL = 43 F_2 plants); error bars are standard errors of the means. F_2 plants with recombination events within the *S1*-*S31* interval (21 plants) are shown individually, while lines with recombination adjacent to the *SP1POL* marker are indicated by the small circles and correspond to those highlighted in **B**. Glume lengths for recombinants are from single plants but correspond to the average length of six glumes from three central spikelets of the main spike. Additional details are presented in Supplemental Data Set S11.

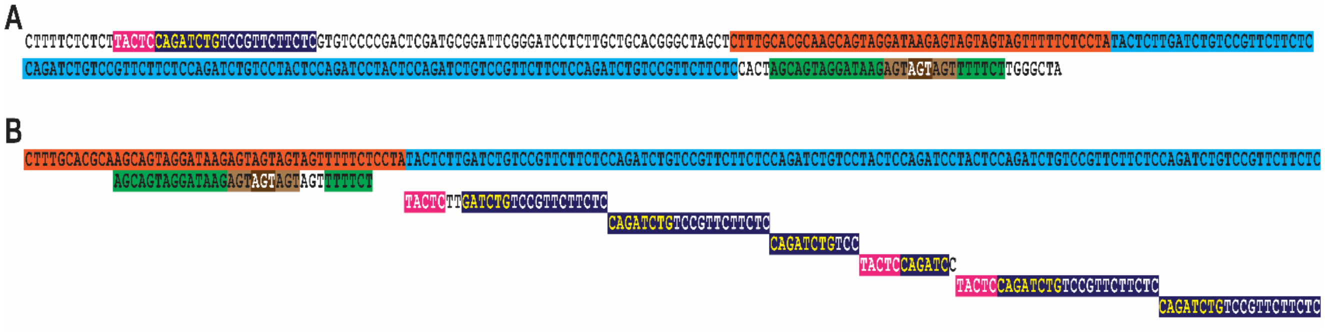

Supplemental Figure S10 (Supports Figure 2, 3): Examination of the 160-bp rearrangement within the *VRT-A2b* allele. (**A**) The 160-bp sequence rearrangement (highlighted in orange and blue) is shown with 5’ and 3’ flanking sequence. The rearrangement has no overall homology to other plant sequences. However, two sequences flanking the rearrangement (highlighted pink/blue and green/brown) occur within the 160-bp rearrangement. (**B**) The 160-bp rearrangement is shown on a single line. Matching flanking sequences are aligned underneath, with nucleotide mismatches displayed in black font with no highlight. The 160-bp sequence can be divided into two sections. The first section (orange) contains a sequence found 3’ of the 160-bp rearrangement (green; AGT units in brown) with a single polymorphism: the 3’ flanking sequence contains three direct repeats of an AGT triplet, while the sequence within the first section has four repeats of this AGT triplet. The second section (blue) consists entirely of tandem copies (full and partial) of a sequence occurring 5’ of the re-arrangement (pink/blue; yellow font represents a palindromic sequence). The entire sequence (25 bp) appears twice within the second section, albeit one copy contains a CA/TT polymorphism. An additional four incomplete copies occur within the second section. These matching sequences account for 145 out of the 160 nucleotides within the *VRT-A2b* rearrangement.

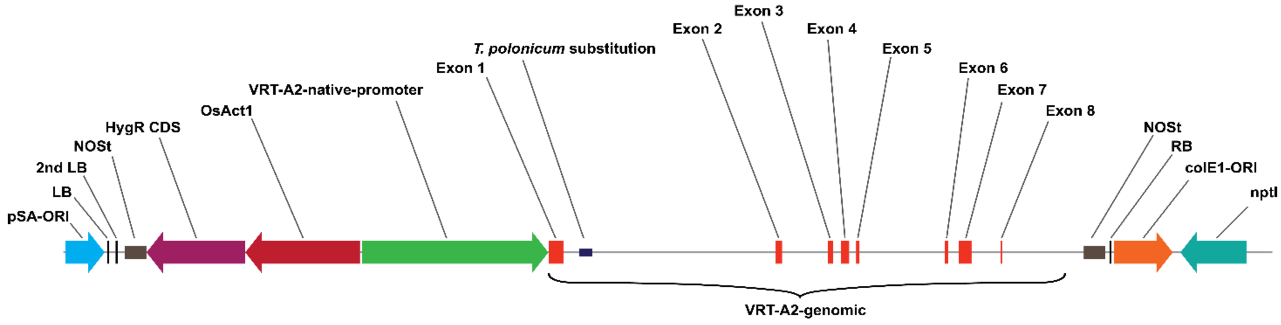

Supplemental Figure S11 (Supports Figure 5): *VRT-A2* complementation construct. The promoter and genomic sequence of *T. polonicum VRT-A2* were cloned into the pGGG-M vector, in opposite orientation to the Hygromycin resistance coding sequence (HygR CDS) driven by the rice *Actin* promoter for selection (Addgene #163703).

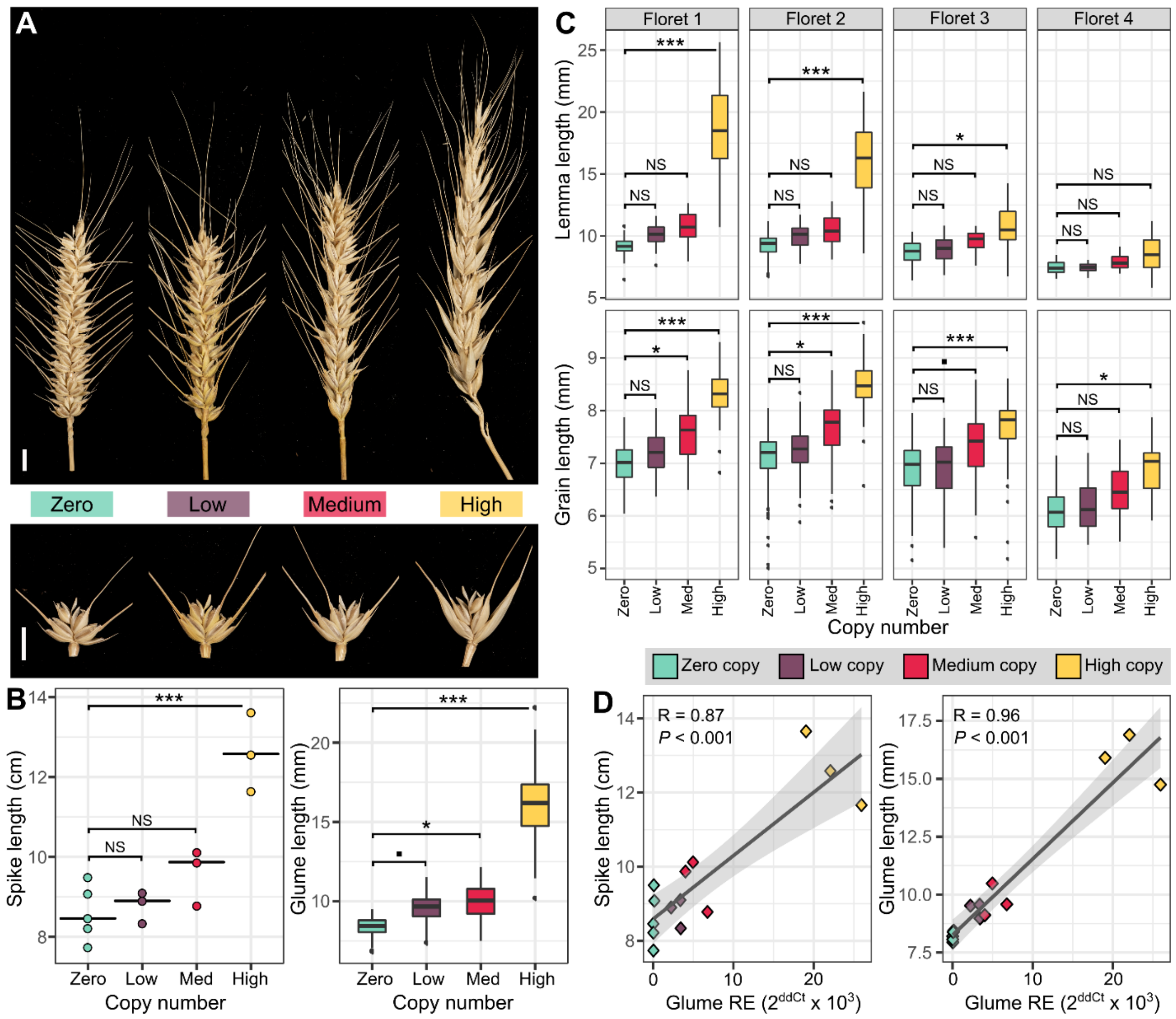

Supplemental Figure S12 (Supports Figure 5): Analysis of T_0 transgenic lines for spike, glume, lemma, and grain length and correlation of spike and glume length with *VRT-A2* expression in glumes. (**A**) Comparison of spikes and spikelets of zero, low (1-2), medium (3-4), and high (9-35) copy number lines (left to right). Notably, spike length increases with copy number, as does glume length. Scale bar = 1 cm. (**B**) Dot and box plots depicting the variation of spike (left) and glume (right) length, respectively, from two tillers of zero (cyan, n=5 plants), low (purple, n=3 plants), medium (red, n=3 plants), and high (yellow, n=3 plants) copy number lines. Horizontal lines represent the median. (**C**) Box plots depicting lemma and grain length for florets 1, 2, 3, and 4 for zero (cyan), low (purple), medium (red), and high (yellow) copy number lines. (**D**) Pearson correlations between *VRT-A2* relative expression in the glume at 21 dpa and spike length (left), and glume length (right). Relative expression shown as 2^{ddCt} x 10^3 (Supplemental Data Set S18). Regression (Dark grey line) and 95% confidence interval (light grey shading) are shown. Data points are coloured according to copy number. Additional correlations in Supplemental Data Set S20. Box plots in (B) and (C) include all subsamples, whereas statistical analyses were performed with mean values. The box represents the middle 50% of data with the borders of the box representing the 25th and 75th percentile. The horizonal line in the middle of the box represents the median. Whiskers represent the minimum and maximum values, unless a point exceeds 1.5 times the inter-quartile range in which case the whisker represents this value and values beyond this are plotted as single points (outliers). Statistical classifications in (B) and (C) are based on Dunnett tests against the zero copy number lines. ▪, $P < 0.10$; *, $P < 0.05$; **, $P < 0.01$; ***, $P < 0.001$.

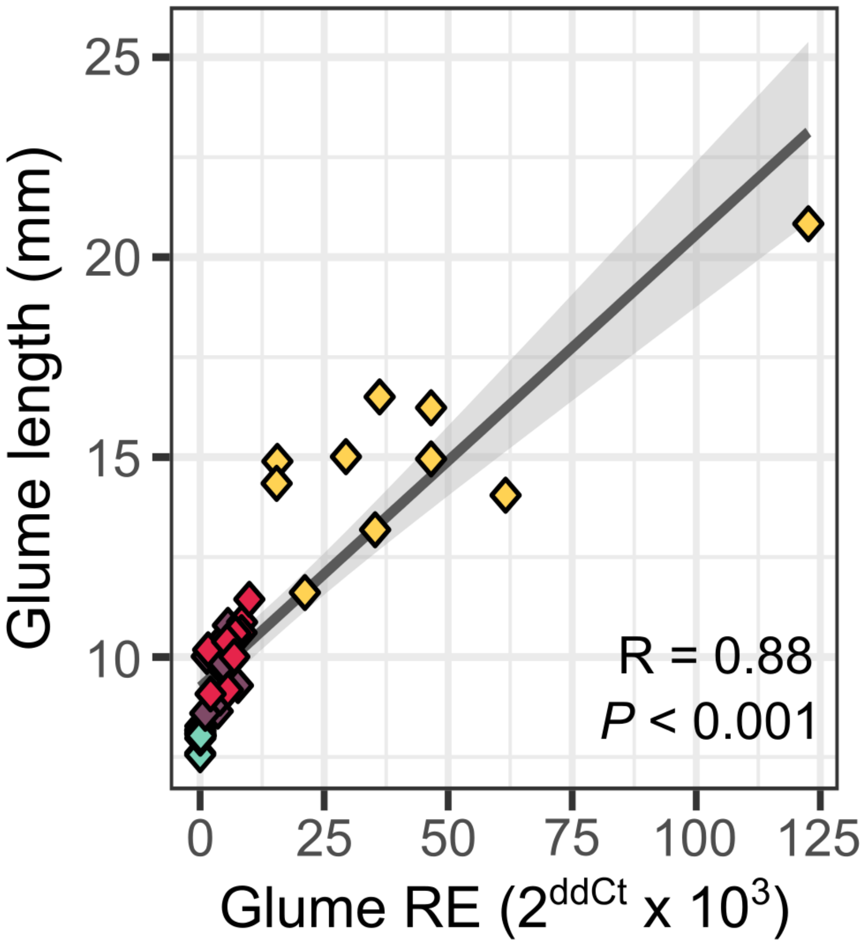

Supplemental Figure S13 (Supports Figure 5): Pearson correlations between *VRT-A2* relative expression in the glume at 14 dpa and glume length including the single outlier value which was not depicted in Figure 5D (n=40 plants). Relative expression shown as 2^{ddCt} x 10^3. Regression (Dark grey line) and 95% confidence interval (light grey shading) are shown. Data points are coloured according to transgene copy number: zero (cyan), low (purple), medium (red), and high (yellow) copy number lines.

References

Felsenstein, J. (1985). Confidence limits on phylogenies: an approach using the bootstrap. Evolution **39,** 783-791.

Saitou, N., and Nei, M. (1987). The neighbor-joining method: a new method for reconstructing phylogenetic trees. Mol Biol Evol **4,** 406-425.

Schilling, S., Kennedy, A., Pan, S., Jermiin, L.S., and Melzer, R. (2020). Genome-wide analysis of MIKC-type MADS-box genes in wheat: pervasive duplications, functional conservation and putative neofunctionalization. New Phytol **225,** 511-529.

Walkowiak, S., Gao, L., Monat, C., Haberer, G., Kassa, M.T., Brinton, J., Ramirez-Gonzalez, R.H., Kolodziej, M.C., Delorean, E., Thambugala, D., ***et al.*** (2020). Multiple wheat genomes reveal global variation in modern breeding. Nature **588,** 277–283

Zuckerkandl, E., and Pauling, L. (1965). Evolutionary divergence and convergence in proteins. In Evolving Genes and Proteins, V. Bryson and H.J. Vogel, eds (Academic Press), pp. 97-166.
