# Supplementary material for: Ectopic expression of Triticum polonicum VRT-A2 underlies elongated glumes and grains in hexaploid wheat in a dosage-dependent manner
Source: Plant Cell. 2021 May 1;33(7):2296–319. doi: 10.1093/plcell/koab119 (PMC8364232; doi:10.1093/plcell/koab119)
Supplement: koab119_Supplementary_Data [file koab119_supplementary_data.zip › tpc.01033.2020-s04.pdf]

Ectopic Expression of *Triticum polonicum VRT-A2* Underlies Elongated Glumes and Grains in Hexaploid Wheat in a Dosage-Dependent Manner

Nikolai M. Adamski, James Simmonds, Jemima F. Brinton, Anna E. Backhaus, Yi Chen, Mark Smedley, Sadiye Hayta, Tobin Florio, Pamela Crane, Peter Scott, Alice Pieri, Olyvia Hall, J. Elaine Barclay, Myles Clayton, John H. Doonan, Candida Nibau, Cristobal Uauy

Corresponding author: Cristobal Uauy (cristobal.uauy@jic.ac.uk).

Review timeline:

**TPC2020-RA-01033**	Submission received:	Dec. 10, 2020
	1st Decision:	Jan 10, 2021 *revision requested*
**TPC2020-RA-01033R1**	1st Revision received:	Mar. 6, 2021
	2nd Decision:	April 4, 2021 *acceptance pending, sent to science editor*
	Final acceptance:	April 17, 2021

REPORT: (The report shows the major requests for revision and author responses. Minor comments for revision and miscellaneous correspondence are not included. The original format may not be reflected in this compilation, but the reviewer comments and author responses are not edited, except to correct minor typographical or spelling errors that could be a source of ambiguity.)

TPC2020-RA-01033 1st Editorial decision – *revision requested* **Jan. 10, 2021**

We have received reviews of your manuscript entitled "Increased and Ectopic Expression of *Triticum polonicum VRT-A2* Underlies Elongated Glumes and Grains in Hexaploid Wheat in a Dosage-Dependent Manner." Thank you for submitting your best work to The Plant Cell. The editorial board agrees that the work you describe is substantive, falls within the scope of the journal, and may become acceptable for publication, pending revision and potential re-review.

We ask you to pay attention to the following points in preparing your revision:

Your manuscript was assessed by three expert reviewers, all of whom found it interesting and of high quality. Although Reviewer 1 asked for higher resolution expression analysis to distinguish between ectopic and higher-level expression of the *VRT-A2b* allele and further analysis of the intronic motifs in *VRT2* transcriptional repression, we decided in the post-review consultation that these points could be dealt with by editing the text. Therefore, we ask you to revise the text to take account of all of the points raised by the reviewers, and particularly to deal with the following issues:

Reviewer 3 finds it surprising that there are no promoter polymorphisms between the alleles and considers this observation critical in the finding that intron polymorphisms are causal. Please confirm this observation and consider the haplotype analysis suggested by the reviewer.

Thoroughly discuss the issues of ectopic versus higher-level expression of *VRT2-A2b* raised by Reviewers 1 and 3.

Discuss the further experimentation suggested by Reviewers 1 and 3 necessary to formally demonstrate the involvement of the intronic motifs in *VRT2* repression.

In Figure 4 A-D please describe the technical and biological replicates used for the RT-PCR analysis. We expect independent biological replicates to have been used.

-- Reviewer comments:

[Reviewer comments shown below along with author responses]

TPC2020-RA-01033R1 1st Revision received **Mar. 6, 2021**

Reviewer comments and **author responses**:

> **We thank all three reviewers for their feedback to our work and their thoughtful comments which have strengthened the manuscript. We also appreciate the post-review consultation which the Editor summarised as follows,**

"Your manuscript was assessed by three expert reviewers, all of whom found it interesting and of high quality. Although Reviewer 1 asked for higher resolution expression analysis to distinguish between ectopic and higher-level expression of the *VRT-A2b* allele and further analysis of the intronic motifs in *VRT2* transcriptional repression, we decided in the post-review consultation that these points could be dealt with by editing the text. Therefore, we ask you to revise the text to take account of all of the points raised by the reviewers..."

> **This feedback has guided some of our responses and edits to the manuscript. Below we present a point-by-point response to the feedback. Note that we refer to <u>line numbers based on the PDF with track changes</u>. In advance, thank you for your time and consideration.**

<u>Reviewer #1</u>

Based on the classical gene cloning and phenotype analysis, the authors show that the gene underlying the long-glume *P1* locus of *T. polonicum* is VRT-A2, one MADS-box transcription factor of the SVP family, which plays pleiotropic functions in grain length, spike length, grain weight and plant height due to increased and ectopic expression of *VRT-A2* in all these tissues of *T. polonicum*. The data in this manuscript are clear and convincible, however, the function of SVP-like gene in promoting glume development has been reported in maize (*ZMM19*), and the elongated glumes and grains conferred by *VRT-A2* don't contribute to yield increase. The interesting part of this study is therefore on understanding why the intron sequence in *VRT-A2a* could repress the expression of *VRT-A2a* in spikelet development, and the correlation between the expression pattern of *VRT-A2b* and spikelet development. I suggest the authors provide more molecular evidence on the following questions and discuss some points to present a reasonable explanation.

> **<u>Response</u>: We agree that these follow-on experiments will be exciting, and these are currently being planned. However, based on the Editor's comments on the outcome of the post-review consultation ("*these points could be dealt with by editing the text*") we have focused on editing sections of the text for the current manuscript.**

The motif 1 and 2 found in the *VRT-A2a* suggested they are binding sites for TFs, and might play important role in regulating the expression patterns of *VRT-A2*, therefore, it's important to confirm these two motifs do function in repressing the *VRT-A2a* expression at the molecular level.

> **<u>Response</u>: As above, and based on the Editor's comments, these specific experiments are beyond the scope of the current manuscript. We have incorporated these ideas into the revised discussion (lines 656 to 665, here and below based on PDF with track changes).**

I am wondering about the correlation between the phenotype and gene expression pattern. The *VRT-A2b* ectopically expressed in the glumes, lemmas, and grains, but why the difference in grain length between P1 NILs is established during mid-grain filling?

> **<u>Response</u>: We show that *VRT-A2b* ectopic expression leads to longer glumes and lemmas compared to wildtype *VRT- A2a* lines. We hypothesise that this leads to a larger floral cavity volume (between lemma and palea) in *VRT-A2b* plants which would allow the grain to develop into a larger volume. We would expect to first detect this effect as the grain expands and this space becomes limited in the wildtype plants due to the physical pressure exerted by the lemma and palea in the more compact wildtype floral cavity. Consistent with this, we first see differences in grain length between NILs during mid-grain filling when grain growth is driven primarily by cell expansion and wildtype grains are approaching their final size. We hypothesise that the physical pressure of lemma/palea at this mid-grain filling stage will inhibit grain expansion in wildtype plants and that this effect will be delayed in *VRT-A2b* plants due to their larger floral cavity volume. We will perform more in-depth studies into this subject for future publications using the transgenic lines.**

Why the expression of *VRT-A2b* in NILs just increase cell length in the centre of the grain? Does *VRT-A2* express higher specifically in the middle part of the grain?

> **<u>Response</u>: We have not conducted the detailed expression analysis in the different sections of the grain. We**

hypothesise that the increase in cell length in the middle of the grain is an indirect effect of the larger cavity volume which manifests itself in these central cells, rather than a direct effect of expression only in the centre. This would be supported by the fact that we see ectopic expression of *VRT-A2b* in floret 4 grains but no effect on the grain size. As mentioned in our previous response, these are really interesting experiments to follow up on. We have included a few sentences in the discussion to highlight the points raised by the reviewer and to highlight the lack of spatial resolution of our current work (lines 508-513).

The expression of *VRT-A2* in floret 4 was similar to that of florets 1+2 based on qRT analysis, therefore, expression pattern analysis of *VRT-A2* in P1 NILs by *in situ* hybridization might give a better explanation for the basipetal gradient effects on the lemma and grain length from basal to apical florets, which also help us to understand the dosage effect of *VRT-A2* in elongated glumes and grain.

Response: We fully agree that *in situ* hybridisations would be very useful to better understand the expression patterns. We hope to pursue these in the short term but as suggested by the Editor and based on the outcome of the post-review consultation, these are currently beyond the scope of this manuscript. We have included comments in the discussion to acknowledge this shortcoming of the current manuscript (lines 547-552).

To confirm the correlation between the *VRT-A2* expression patterns with the long-glume phenotypes of *T. polonicum*, the authors transformed the hexaploid accession 'Fielder' using the genomic *T. polonicum VRT-A2b* allele (5591 bp), and found ectopic expression of *VRT-A2* leads to phenotypic effects in a dosage dependent manner. However, there has only one copy of *VRT-A2b* in the P1POL plant, I am afraid the copy number can't explain the correlation since those lines with low copy numbers didn't present significant differences compared to the wildtype.

Response: Our statements on dosage-dependent effects are based (to a large extent) on the correlation between *VRT-A2* expression and the magnitude of phenotypic effects on several traits. These are depicted in Figure 5D (glume and spike length) and Supplemental Table 18 (lemma, palea, and grain length). We think that evaluating these relationships based on expression data, rather than the categorical copy number, is a more adequate representation of the biology. While copy number and expression are certainly related, in Supplemental Table 18 we show correlations between expression levels in different tissues with the phenotypic effects on the multiple traits listed above. In the original manuscript we found strong correlations between *VRT-A2* expression levels in the glume and spike length (R=0.87), glume length (R=0.95), and floret 1 lemma length (R=0.94), palea length (R=0.91) and grain length (R=0.90); all of which are highly significant *P* <0.0001. These results are also consistent with Li et al., 2020 *bioRxiv* who also show a dosage relationship between *VRT-A2* overexpression (using maize *Ubiquitin* promoter) and the magnitude of phenotypic effects in glume length.

Having said this, we also acknowledge that in the initial characterisation of the transgenic lines our level of replication was not as high as we would have liked. In the results section of the initial submission we did point out this shortcoming by stating, "Due to the relatively small sample size for each category (N=3 to 5 T_0 plants), we did not detect significant differences between zero copy number lines and low copy number lines for any of the traits, although glume length increased by 14.1% (*P* <0.08)."

We have now incorporated additional data and analyses based on a larger number of T_1 plants. For this revised version we have analysed 40 additional plants: five plants each of eight different transgenic events (two zero copy, two low copy, two medium copy and two high copy number lines). The analysis of the T_1 plants has confirmed our initial results in the T_0 generation and shows very significant correlations between *VRT-A2* expression and the magnitude of the phenotypic effects. In this new dataset, we find significant correlations between *VRT-A2* expression levels in the glume and glume length (R=0.87), floret 1 lemma length (R=0.89), palea length (R=0.76); all of which are highly significant *P* <0.0001. For grains, we also find significant correlations between *VRT-A2* expression levels in grain 2 and its length (R=0.45; *P*<0.005). In addition, we quantified expression of *VRT-A2* in the rachis and also found significant correlations between its expression and spike length (R=0.72; *P* <0.0001). These results strongly support the conclusion of a dosage-dependent response. The updated results based on the T_1 generation are presented from line 393 onwards, as well as in the updated Figure 5, and Supplementary Tables 16-18.

We also performed ANOVA between the zero, low, medium, and high copy number lines. Here we find significant differences between zero and low number copy lines for glume length (20%; *P*=0.03) which is the main phenotypic effect described for *P1*. These effects become increasingly more significant in the medium (28%, P=0.002) and high (89%, P<0.0001) copy number lines. Similar increases in magnitude and significance are also evident for spike,

lemma, palea, and grain length with increasing copy number, albeit the low-copy number category is not always significantly different to the zero copy lines. These new results support our previous data, and together with the correlation analysis (based on *VRT-A2* expression rather than copy number), suggest a dosage-dependent effect of *VRT-A2* on multiple spike phenotypes in polyploid wheat.

Based on the expression pattern analysis of *VRT-A2* in P1POL NIL, could ectopic expression of *VRT-A2b* in spikelet organs be enough to promote its elongation? In the ectopic expression lines, why different organs showed a distinct effects, especially in paleae?

<u>Response</u>: In our original discussion we provided a possible explanation for this phenomenon, "We hypothesise that SVP is able to compete more strongly with protein complexes required for glume and lemma development (as shown in Li et al. (2020)) and gradually less so with those protein complexes involved in palea development, which include additional MADS-box proteins (e.g. *ALG6-like* genes; (Reinheimer and Kellogg, 2009)). This would explain the dosage-dependent response observed in our study and why we observe the strongest effects in outer and early established organs (e.g. glumes and lemmas) while later developing/differentiating organs (e.g. paleae) are affected only in lines with the highest *VRT-A2* expression (Figure 5, Supplemental Table 16).".

We have now written a new paragraph in the discussion to explain the phenotypic gradients observed in the study more clearly (lines 561 to 571). We have also expanded the section mentioned above to address this, and reviewer three's, comments (see lines 625-627).

<u>Reviewer #2</u>

1. Please re-consider the title. The most exciting aspect of the paper is the identification of a gene that influences grain size wheat and that also defined a wheat subspecies in the era of descriptive classification. The short title is better than the longer one, which is technical by comparison. Suggest a title along the lines of "...P1 gene...increases grain size and defines *Triticum polonicum* subspecies...arises from increased expression of VRT2".

   <u>Response</u>: Thank you for the suggestion. We discussed amongst the authors and based on other reviewers' comments we have adjusted the title slightly but maintained its general structure. We considered that the *P1* gene nomenclature might be less well-known to a general audience and we wanted to maintain the dosage effect within the title. We have added an additional keyword to ensure that the manuscript is also found by those looking for the *P1* gene.

2. Line 31 Important determinant of seed/grain yield.

   <u>Response</u>: We have changed as suggested (line 30, here and below based on PDF with track changes).

3. Line 191. From reading the methods and materials it is clear how the team were able to target a narrow chromosome region for fine mapping. It would be good to outline that process in the main text - high-density SNP array genotyping of the NILs followed by exome capture. Also, in the methods and materials, these details could shift from line 731 to line 722, to precede the outline of the fine mapping process.

   <u>Response</u>: We have added some additional text in the results section to clarify the screen performed to identify the recombinant plants (line 198). We did not want to delve too deeply, however, into the origin of the markers, etc as we used relatively well-established approaches which are all detailed in the methods. We have re-ordered the sections in the methods as suggested by the reviewer (see line 819-840). They make more sense now: thank you.

4. Line 219. Can this be reworded slightly to emphasise the loss of a 563-bp segment? Later in the manuscript it becomes important when the potential regulatory motifs are discussed, but it could be emphasised more here that a segment of DNA is lost and replaced by repetitive sequences.

   <u>Response</u>: We have reworded this section to emphasise the loss of the 563-bp segment as suggested by the reviewer and the haplotype analysis as suggested by reviewer 3 (lines 223-234).

5. Figure 2C. The coloured boxes included as a key in the glume length panel - these need to be defined and possibly placed outside the box? (or place a box around the key and keep as an inset). At the moment they first appear to be data points.

   Response: As suggested, we have placed a box around the key to make it more distinct.

6. Figure 4E. Define MB and LB (mid-boot and late boot).

   Response: We have added these definitions as suggested (line 364).

7. Line 585… is caused by the sequence insertion in the *VRT-A2b* allele.

   Response: We have amended the sentence to "…is caused by the 160-bp sequence substitution of the *VRT-A2b* allele" (see line 653).

8. I wish the authors the best of luck with this research.

   Thank you!

Reviewer #3:

Major comments

1. I was very surprised that sequencing of nearly 10kb of both coding and non-coding regions of the *VRT-A2b* allele showed only the intron polymorphism relative to the RefSeqv1.0. This is truly remarkable, and only would make sense to me if the reference line had the ancestral haplotype that gave rise to *VRT-A2b*. Is it possible to determine the ancestral haplotype that gave rise to this variant? Another possibility is that this locus has significantly reduced sequence polymorphism associated with a selective sweep or domestication bottleneck. Is there indeed reduced nucleotide polymorphism in wheat compared to other neutral loci?

   Response: We agree that this is in fact surprising. In the original submission we included Supplemental Table 10 which showed the haplotype analysis based on Sanger sequencing of the POL NILs and the full genome sequence of 19 hexaploid and tetraploid wheat accessions, including wild emmer. We find that Chinese Spring and Norin61 both share the same overall haplotype with *T. polonicum*, apart from the 563-bp intron 1 re-arrangement. There are an additional four *VRT-A2* haplotypes across the wider interval (including promoter, and coding/intron sequence) which include multiple additional SNPs and indels, both in the promoter and the coding/intron sequences. Two of these other haplotypes also comprise both tetraploid and hexaploid accessions. Across haplotypes, the only consistent polymorphism with the *P1^{POL}* sequence is the substitution of the 563-bp intron-1 sequence for the 160-bp sequence.

   To further document this, we have now included the analysis of an additional F_2 population between Chinese Spring and the *P1^{POL}* NIL. These lines share the same *VRT-A2* haplotype, except for the intron 1 sequence substitution. The glume length phenotype matched perfectly with the *VRT-A2* allele within these lines, providing additional evidence that the substitution of the 563-bp sequence in intron 1 by the 160-bp sequence is the causal polymorphism. The haplotype analysis and this additional data is discussed in the results section (line 223 to 233, here and below based on PDF with track changes) and in Supplemental Table 11 and Supplemental Figure 9.

   In addition, we have now deposited in GenBank the sequences of the multiple accessions of *T. polonicum* (n=6), *T. petropavlovskyi* (n=2) and *T. aestivum* 'Arrancada' (n=4) which we sequenced. We have also indicated in Supplemental Table 13 the exact length of the promoter, genomic, and 3' UTR sequence which was sequenced and deposited.

2. The phenotypic analysis of the recombinants used for fine mapping (Fig. 2b) suggest a potentially complex QTL controlling glume length. In particular, lines R4-R7 seem to have significantly smaller glumes compared to the NIL and R2-R3. This reduction in glume length seems to be approximately on the order of what you might expect for a heterozygote. Notably, these lines all contain recombination events in the 5' promoter region, which could be suggestive of another *cis*-element that, in combination with the intron variant, is required for the complete glume phenotype. Fine mapping of the *cis*-element responsible for the tb1 QTL in domesticated maize (Studer et al., 2011

doi: 10.1038/ng.942) was also shown to be complex. Is the reduced glume length of lines R4-R7 relative to the NIL and R2-R3 significant? If so, I think the authors should consider the possibility that other regulatory elements may contribute to the glume phenotype.

Response: Thank you for raising this really interesting point. The glume phenotype is certainly intermediate when analysing R2-3 vs R4-7, although other phenotypes (such as TGW) were non-significant in the same analysis.

However, the reviewer's point is well made. We have included a statement in the results section to consider the possible presence of additional genetic elements that could modulate the *magnitude* of the *P1* locus glume phenotype (lines 204 to 206).

3. While the authors make a strong case that the intron polymorphism is causative, the ideal confirmation would be CRISPR-induced deletion of just the candidate intron region from wt to re-capitulate phenotype. I understand that this approach is likely to be complicated by the tetrapoloid genome and beyond the scope of this paper, so I'm not expecting that it could be done in a revision. Rather I just wanted to communicate what I see as the best possible confirmatory evidence. An alternative confirmation that the change in expression is caused by a cis-regulatory element could be done with allele specific expression using a heterozyogote with a wt allele that has a polymorphism in the coding sequence. If the *VRT-A2b* allele has significantly increased expression relative to the wt allele in the same plant, that would provide compelling evidence that indeed a *cis*-acting element is responsible, although the approach could not definitively confirm that it was the candidate site in the intron.

Response: We agree that these experiments will be of interest. The Editor commented that based on the post-review consultation, "*these points could be dealt with by editing the text*". We have therefore incorporated additional statements throughout the discussion to address these points, for example lines 656 to 665.

Minor comments

1. Increased expression and ectopic expression are not always distinct categories. The resolution afforded by RT-PCR can't rule out the possibility that increased levels observed for *VRT-A2b* is not actually caused by ectopic expression in novel regions of the tissue sampled. If the wt allele is expressed in a small domain of the meristem, but the VRT-A2b allele expands its domain in the meristem (without increasing the local level of expression) this would give the appearance of increased levels, but would be more appropriately interpreted as ectopic expression. Distinguishing among these possibilities would require detailed in situ hybridization, which I do not think is necessary. Rather, I would suggest massaging the language to acknowledge that subtle point.

Response: We completely agree with this point. We have incorporated an explicit statement in the discussion into the issue of ectopic versus higher expression (lines 547 to 552). We have also reworded the title to remove the concept of 'higher' expression. Similarly, we have modified language accordingly throughout the text to account for this issue. When describing the actual qRT-PCR results we maintained the higher/increased nomenclature, but when *interpreting* the expression results or providing a summary/take-home message we have only used ectopic expression

2. I'm not familiar with the convention in wheat, but if *T. turgidum* ssp. *polonicum* is the current taxonomy for this line, it doesn't seem correct to refer to it as *T. polonicum* throughout the paper as it implies that *polonicum* is the species epithet. Related to this, it seems inappropriate to refer to the glume phenotype or the associated mutation as a "species defining polymorphism" (line 484, 633). First, because this is a subspecies, and second because a single, simple genetic polymorphism is usually not sufficient to define a species.

Response: There does not seem to be clarity on this point. Linnaeus classified it as a distinct species and subsequent studies have either maintained its species name as *Triticum polonicum*, while others have opted for the subspecies name *Triticum turgidum* ssp *polonicum*. Often the names are considered as synonyms. To avoid over-hyping the results we agree with the reviewer that the more conservative "sub-species defining polymorphism" phrasing is more adequate. We have therefore rephrased this throughout the revised manuscript. However, to avoid the extended *Triticum turgidum* ssp *polonicum* nomenclature across the text, we have defined *T. polonicum* as a synonym at the start to facilitate reading of the manuscript.

3. The paragraph from line 516-528 was confusing to me. I don't understand how MADS box protein complexes can explain the clear apical basal phenotypic gradient in the spikelet. As I understand it, the floral MADS-box complexes do not significantly change along this axis (i.e. each subsequent floret should have the same array of MADS-box protein complexes), although they do change across the organs of a floret.

   Response: We agree with the reviewer that this discussion was not as clear as it could have been. We have now introduced a new paragraph to outline the two phenotypic gradients (apical-basal along the spikelet and inner-outer within floret) more clearly (line 561 to 571). We have also adjusted the paragraph mentioned here (now lines 625 to 627) to state our thinking behind the apical-basal gradient more explicitly.

4. I couldn't understand the purpose of the sentence (line 584-5): "We cannot exclude the possibility that misexpression of *VRT-A2* is caused by the *VRT-A2b* allele".

   Response: We wanted to recognise the alternative explanation that the *VRT-A2b* 160-bp rearrangement in intron 1 could lead to the misexpression of the gene. We have reworded and expanded this sentence. We also placed these sentences within the context of the need for gene editing of the motifs for additional experimental evidence (line 653 to 665).

TPC2020-RA-01033R1 2nd Editorial decision – *acceptance pending* April 4, 2021

We are pleased to inform you that your paper entitled "Ectopic Expression of *Triticum polonicum VRT-A2* Underlies Elongated Glumes and Grains in Hexaploid Wheat in a Dosage-Dependent Manner" has been accepted for publication in The Plant Cell, pending a final minor editorial review by journal staff.

All reviewers found the revisions satisfactory and recommended acceptance of the manuscript. While Reviewers 1 and 2 asked for no further changes, Reviewer 3 mentioned again the use of the word ectopic in the title and results. The reviewer points out that the data do not distinguish between "overexpressed" and "ectopic" and that this is clearly stated in the Discussion section but not the other sections. During the final editing you might wish to make this distinction clearer in the earlier sections of the paper.

Final acceptance from Science Editor April 17, 2021
